# Do Better Quality Embedding Potentials Accelerate the Convergence of QM/MM Models? The Case of Solvated Acid Clusters

**DOI:** 10.3390/molecules23102466

**Published:** 2018-09-26

**Authors:** Junming Ho, Yihan Shao, Jin Kato

**Affiliations:** 1 School of Chemistry, University of New South Wales, Sydney, NSW 2052, Australia; jinkato.jk@gmail.com; 2 Department of Chemistry and Biochemistry, University of Oklahoma, Norman, OK 73019, USA; yihan.shao@ou.edu

**Keywords:** QM/MM, ONIOM, effective fragment potential, semi-empirical methods, convergence

## Abstract

This study examines whether the use of more accurate embedding potentials improves the convergence of quantum mechanics/molecular mechanics (QM/MM) models with respect to the size of the QM region. In conjunction with density functional theory calculations using the ωB97X-D functional, various embedding potentials including the TIP3P water model, the effective fragment potential (EFP), and semi-empirical methods (PM6, PM7, and DFTB) were used to simulate the deprotonation energies of solvated acid clusters. The calculations were performed on solvated neutral (HA) and cationic (HB^+^) acids clusters containing 160 and 480 water molecules using configurations sampled from molecular dynamics simulations. Consistently, the ωB97X-D/EFP model performed the best when using a minimal QM region size. The performance for the other potentials appears to be highly sensitive to the charge character of the acid/base pair. Neutral acids display the expected trend that semi-empirical methods generally perform better than TIP3P; however, an opposite trend was observed for the cationic acids. Additionally, electronic embedding provided an improvement over mechanical embedding for the cationic systems, but not the neutral acids. For the best performing ωB97X-D/EFP model, a QM region containing about 6% of the total number of solvent molecules is needed to approach within 10 kJ mol^−1^ of the pure QM result if the QM region was chosen based on the distance from the reaction centre.

## 1. Introduction

Hybrid quantum mechanics/molecular mechanics (QM/MM) methods were first proposed some 40 years ago and have since been further developed and successfully applied to study the structure, energetics, and dynamics of a variety of chemical and biochemical processes [1,2,3,4,5]. The basic idea behind these methods is to partition the system into two or more layers that are treated at different levels of accuracy and computational cost, thereby enabling the application of quantum chemical calculations to much larger systems. The impact of these methods and their contribution to chemistry were recognised by the 2013 Nobel Prize in Chemistry [6].

Despite the widespread use of these methods, it was not until recently that computational chemists were able to systematically examine the convergence of these models with respect to the size of the QM region. Notably, recent advances in computer architectures and quantum chemistry algorithms have made it possible to carry out single point energy calculations on very large molecular systems. Harnessing these advances, numerous groups have examined the rate of QM size convergence for QM/MM calculations of properties ranging from NMR shielding constants [7,8], absorption spectra [9,10,11] to barrier heights [12,13,14,15] and redox potentials [16].

An interesting finding from some of these studies is that QM/MM calculations converge rather slowly with respect to the size of the QM region. For example, Martinez and Kulik examined the methyl transfer reaction in catechol-*O*-methyl transferase and found that around 500–600 QM atoms are needed to approach the QM/MM asymptotic limit if the residues were chosen simply by distance from the reaction centre [13]. Similarly, a recent study concluded that a total of ca. 1150 QM atoms is needed for a reliable description of proton transfer processes within DNA [17]. However, there are also studies of the same systems which showed rapid convergence if the QM region was selected appropriately based on chemical intuition [14,18].

When one considers the number of parameters involved in a typical QM/MM simulation, it is not surprising that studies of the same system carried out by different research groups can sometimes lead to diverging conclusions. In a typical QM/MM simulation, one needs to decide on the choice of QM/MM method (e.g., subtractive or additive), QM theory and MM force field, embedding scheme (mechanical or electronic embedding), treatment of boundary atoms, effect of geometry optimisation, and configurational sampling. The array of possible QM/MM approximations makes it challenging to directly compare these models and evaluate the impact these parameters have on their accuracy. Carefully designed studies are needed to properly address the sensitivity of QM/MM models to these parameters. For example, a recent study by Major and co-workers revealed that QM/MM single-point calculations on structures minimised at the MM level of theory led to greater size dependence of the QM region for proton transfer in DNA compared to QM/MM optimised structures [18]. Ryde and co-workers have also evaluated the errors associated with various treatments of boundary atoms and embedding schemes [19]. The authors found that mechanical embedding (ME) actually gave better results than electronic embedding (EE) for proton transfer in [Ni,Fe] hydrogenase, while Roßbach and Ochsenfeld discovered that the EE scheme led to a six-fold reduction in QM region for size convergence compared to ME for proton transfer in DNA [17]. In the EE scheme, the electrostatic interactions between the QM and MM subsystems are treated at the QM level by including the atomic charges of the MM subsystem in the QM Hamiltonian, and is presumably more accurate because it allows the QM wavefunction to be polarised.

The aforementioned studies suggest that the optimal QM/MM procedure will likely depend on the specific properties investigated and the nature of the system, i.e., environment around the reaction centre. So far, most QM/MM studies are focused on a specific biochemical system, and when comparisons are made between different types of systems, e.g., proton-transfer in DNA versus in an enzyme, it is unclear if the good performance of a specific modelling protocol can be carried over to other systems. To this end, this study aims to examine whether the use of more sophisticated embedding potentials improves the accuracy and accelerates the QM region size convergence in QM/MM models. The potentials range from fixed charge TIP3P water model [20], to polarisable effective fragment potential (EFP) and semi-empirical PM6 [21], PM7 [22], and density functional tight-binding (DFTB) [23] methods. The EFP model is a QM-based force field that provides an accurate description of Coulombic interactions, polarisation, exchange repulsion, dispersion, and charge transfer [24,25]. This study will focus on calculations of deprotonation energies in solvated acid clusters which are ideal because one can easily probe the performance of QM/MM models under different circumstances, e.g., the chemical environment and/or the nature of the reaction centre can be tuned by changing the solvent and/or acid. It also avoids complications associated with the treatment of boundary atoms since the QM/MM boundaries do not cut across covalent bonds.

This paper is organised as follows: we first describe the test set of neutral and cationic acids and our computational procedures. This is followed by results and discussion, where we compare the convergence behavior of various QM/MM methods with respect to the choice of embedding potentials and schemes. The sensitivity of these models with respect to these parameters, and how it varies between the two classes of acids, are discussed.

## 2. Experimental Design and Methods

**Figure 1:**
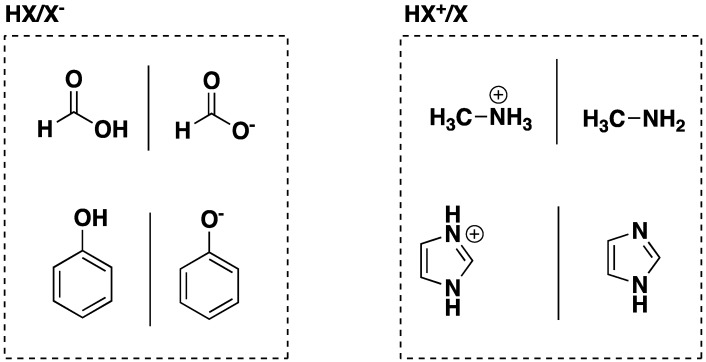
Structures of acids examined in this work.

In this study, hybrid QM/MM and QM/QM′ methods were used to calculate the deprotonation energies of neutral (HA/A^−^) and cationic acids (HB^+^/B) in water clusters containing up to 480 solvent molecules. In this notation, MM and QM′ refer to the embedding potential used to model the environment around the QM region. The test set consists of two examples from each class (HA = HCOOH and C_6_H_5_OH) and (HB^+^ = CH_3_NH_3_^+^ and H-imidazole^+^) as shown in Figure 1. Within each class, the acids differ in their extent of charge delocalisation, i.e., HCOO^−^ and CH_3_NH_3_^+^ represent relatively charge-concentrated ions while C_6_H_5_O^−^ and protonated imidazole are significantly more diffuse (delocalised). Using the HA/A^−^ system as an example, the deprotonation energy is defined in this study as the energy change (at 0 Kelvin without zero-point vibrational correction) associated with the following reaction:

^−^HA(H_2_O)*_m_* → A^−^(H_2_O)*_m_* + H^+^

Solvated clusters of two sizes were examined (*m* = 160 or 480), and configurational sampling was carried out using molecular dynamics simulations of the acid solvated in a periodic box of water molecules. The solvated clusters were obtained by considering the *m* nearest solvent molecules from the solute. For a given snapshot, the structure of the deprotonated form is generated by deletion of the acidic proton, whilst the coordinates of the remaining atoms are unchanged. Thus, the ‘deprotonation energy’ simulated in this study mimics that of a vertical process (or sudden deprotonation), in contrast to adiabatic deprotonation. It should be emphasised that the aim of this study was to examine how the accuracy and convergence of hybrid models (relative to pure QM data of the full system) were affected by the choice of embedding potential, and not to reproduce the experiment.

**Figure 2:**
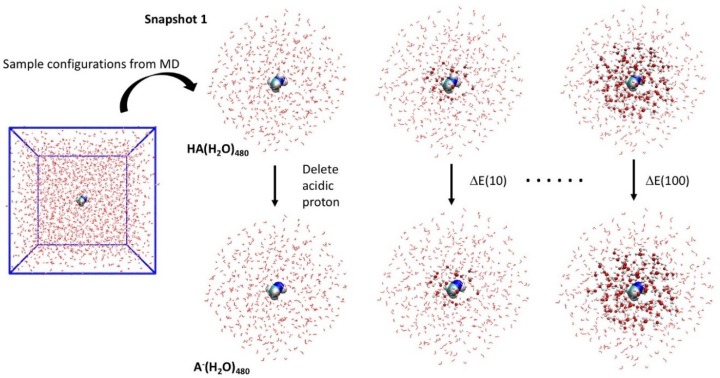
Snapshots of the solvated clusters (*m* = 480) are collected from a molecular dynamics simulation of the solute in a periodic box of water. For a chosen snapshot, a series of quantum mechanics/molecular mechanics (QM/MM) models are set up by radially increasing the size of the QM region (balls and sticks) from the reaction centre. ΔE(*m*) refer to the QM/MM simulation with *m* water molecules in the QM region. Errors in QM/MM models are measured from absolute deviations from the pure QM calculation of the full system, ΔE = ΔE (480).

A total of 30 snapshots were randomly collected from a 4 ns production run. Figure 2 describes how the QM region size convergence of the QM/MM and QM/QM′ models is evaluated. For each snapshot, a fully QM single-point calculation was carried out to obtain the reference deprotonation energy (ΔE). To examine the QM region size convergence of the hybrid models, the QM region was radially expanded to include an increasing number of solvent molecules based on distance from the reaction centre (in increments of 10 or 30). ΔE(*m*) refers to a QM/MM or QM/QM′ simulation with *m* water molecules in the QM region, and the error in the model is calculated as |(ΔE − ΔE(*m*)|. All reported errors in the QM/MM approximations correspond to averages over the 30 snapshots.

In the QM/MM and QM/QM′ models, density functional theory calculations using the ωB97X-D functional and 6-31G(d) basis set were used to model the QM subsystem, whilst the low-level or MM subsystem was simulated using the TIP3P water potential [20], the effective fragment potential (EFP) [24,25,26], as well as semi-empirical PM6 [21], PM7 [22], and DFTB [27] methods. The QM-only approach corresponds to calculations based only on the QM subsystem. For the QM/TIP3P model, both mechanical and electronic embedding (ME and EE) schemes were also considered. With the exception of the QM/EFP method (which is an additive QM/MM approach as implemented in Q-Chem), we used the ONIOM method [28,29,30] which is a subtractive scheme as implemented in Gaussian. In the additive scheme, the energies of the QM and MM subsystems and interactions between the two systems are added to obtain the total QM/MM energy of the whole system, whilst in a subtractive scheme, the corresponding energy is evaluated as the QM energy of the QM subsystem plus the MM energy of the full (QM + MM) system minus the MM energy of the QM subsystem—see Equation (1).

For the ONIOM(ωB97X-D/6-31G(d):MM) calculations, the general Amber force field [31] was used to simulate the solute together with the TIP3P water model. The atomic charges for the solute were obtained using the restrained electrostatic potential (RESP) method [32] at the HF/6-31G(d) level using the Antechamber program [33]. For the DFTB method, we used the DFTBA version with analytic expressions for the matrix elements rather than tabulated ones [27], and for the PM7 method [22], we used the version by Throssel and Frisch that includes numerically stable hydrogen bond corrections [34]. Because the built-in EFP parameters for water in Q-Chem are based on the MP2/cc-pVTZ optimised geometry, the EFP parameters (HF/6-31 + G(d)) for water were recomputed for TIP3P geometry using GAMESS [35], and all QM/EFP calculations were performed using Q-Chem [36,37].

All ONIOM calculations were performed using the Gaussian16 [34] program, whilst the molecular dynamics (MD) simulations were carried out using the NAMD program [38] in conjunction with the CHARMM [39] and CGENFF [40] force fields. In the MD simulation, the acid was placed at the origin (centre) of a 4 nm periodic box of TIP3P water molecules. The configuration of each solvated acid was first relaxed by energy minimisation for 1000 steps, followed by equilibration for 1 ns, and a 4 ns production run. During the simulation, a harmonic constraint (1.0 kcal mol^−1^ Å^−2^) was applied to restrain the centre of mass of the acid to the origin. The simulations were performed for an isothermal-isobaric (NPT) ensemble, using a 1 fs integration time step and rigid bonds for water. The Langevin thermostat was used to maintain constant temperature of 300 K, and the Nosé-Hoover method was used to maintain constant pressure at 1 bar. Long-range electrostatic interactions were calculated with the particle-mesh Ewald algorithm, the Lennard-Jones interactions were truncated at 12 Å, and a switching function was applied at 10 Å. Visualisation and analysis of the MD trajectories were performed using the VMD program [41]. In-house Tcl and Perl scripts were used to extract geometries of the solvated acid clusters and for generating the ONIOM input files.

The QM/MM (or QM/QM′) energy expression for the ONIOM-ME method with *m* water molecules in the QM region is shown in Equation (1):

E(*m*) = E^H^(*s* + *m*) + E^L^(full) − E^L^(*s* + *m*) (1)

where *s* + *m* refers to the solute and the number of water molecules in the QM subsystem, ‘full’ refers to the entire system, and the superscripts ‘H’ and ‘L’ indicate that the terms are calculated using a high and low-level method respectively, i.e., H = QM and L = QM′ or MM. The same expression may be used for ONIOM(QM:MM)-EE calculations, but in this case, the MM point charges are included in the QM term, i.e., E^H^(*s* + *m*) becomes E^H+ptchg^(*s* + *m*), and setting the charges of the QM subsystem to zero in the MM calculations [42].

**Figure 3:**
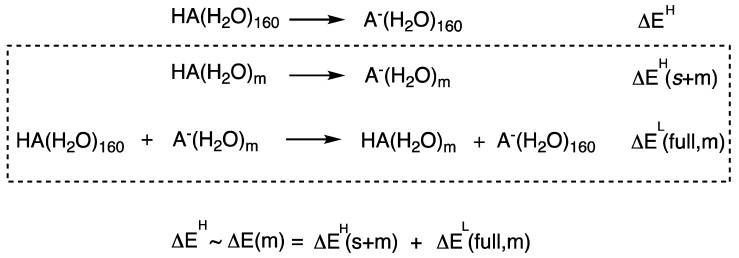
Illustration of the ONIOM-ME approach for calculating deprotonation energies.

Figure 3 provides a pictorial explanation of the ONIOM-ME method for the calculation of deprotonation energy for an acid solvated by 160 water molecules. As shown, ΔE(*m*) is the ONIOM approximation to the QM calculation of the entire system ΔE^H^, and is obtained as the sum of two reaction energies ΔE^H^(*s* + *m*) and ΔE^L^(full, *m*), where *m* is the number of water molecules in the QM region. Note that ΔE^L^(full, *m*) is formally an isodesmic (proton exchange) reaction and therefore would benefit from cancellation of errors associated with the lower-level method ‘L’ or embedding potential. As such, the agreement between the full QM (ΔE^H^) result versus the QM/MM approximation (ΔE(*m*)) is directly measured by the difference between ΔE^L^(full, *m*) and ΔE^H^(full, *m*). 

## 3. Results and Discussion

**Figure 4:**
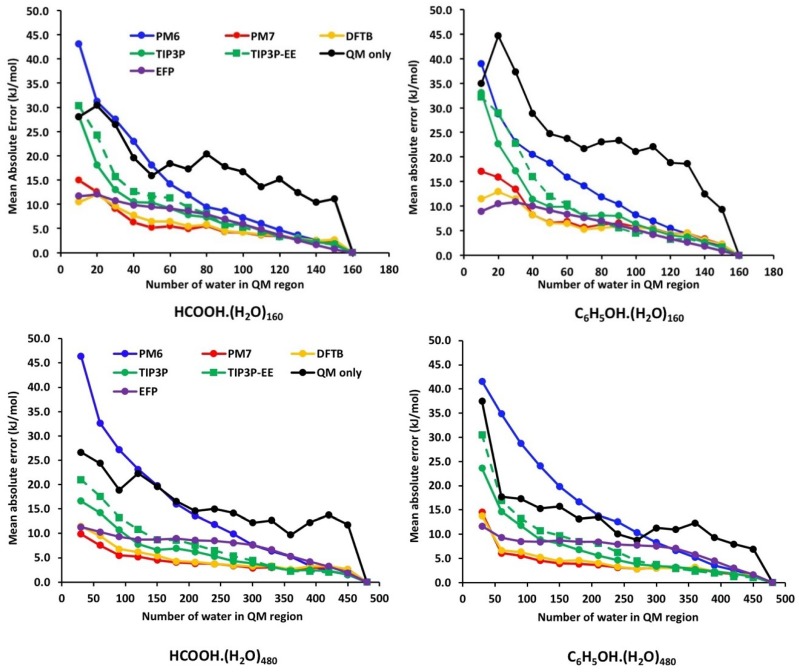
Error convergence profiles for the various QM/MM and QM/QM′ models as a function of QM region size for 160-water cluster (**top**) and 480-water cluster (**bottom**) of neutral acids.

**Figure 5:**
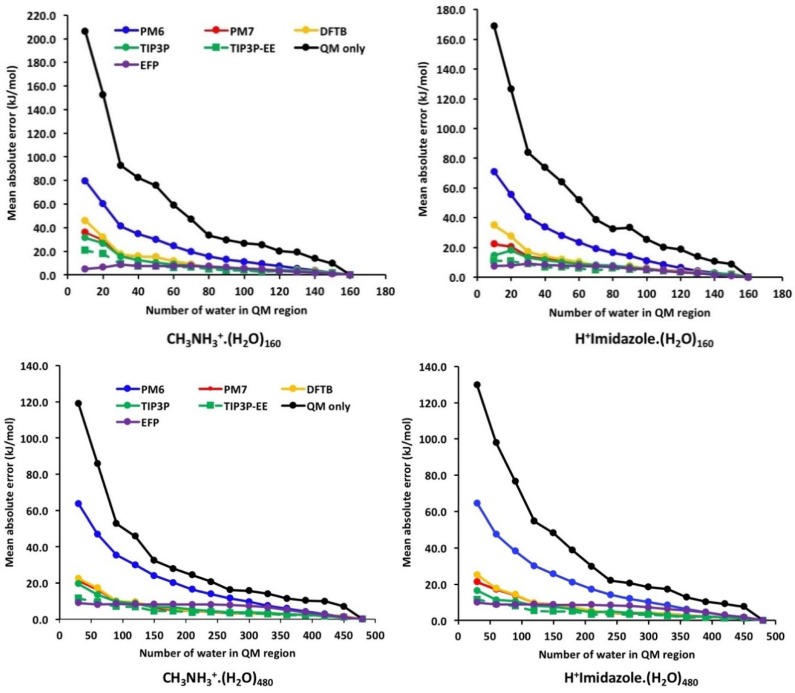
Error convergence profiles for the various QM/MM and QM/QM′ models as a function of QM region size for 160-water cluster (**top**) and 480-water cluster (**bottom**) of cationic acids.

Figure 4 and Figure 5 show the error convergence profiles for the QM/MM and QM/QM′ models as a function of QM region size for 160-water and 480-water clusters of neutral and cationic acids (Tables S1–S8 in Supplementary Materials). The vertical axis represents the absolute error |(ΔE − ΔE(*m*)| in the approximation relative to the pure QM result of the full system, averaged over 30 snapshots from MD. With increasing QM region size (moving along the horizontal axis), all hybrid models approach the full QM result, and the error tends to zero. Because the error convergence profiles for the 160 and 480 water clusters are qualitatively very similar (with the exception of C_6_H_5_OH/C_6_H_5_O^−^ where there is an inversion between QM-only and PM6 for the two clusters), we will base most of our discussion on the 160-water cluster data. For the purpose of our discussion, we refer to the rate of convergence to mean how quickly the errors decay to below a cut-off value of 10 kJ mol^−1^.

For both HA/A^−^ systems (Figure 4), the performance of the EFP method is comparable if not slightly better than the semi-empirical PM7 and DFTB methods, particularly for the smallest QM region (*m* = 10) where its mean absolute errors are 11.7 and 8.9 kJ mol^−1^ for the HCOOH/HCOO^−^ and C_6_H_5_OH/C_6_H_5_O^−^ systems, respectively. In the 480-water cluster models, the EFP retains a similar level of accuracy; however, the errors remain relatively insensitive to the QM region size, and only start decaying towards zero after about 300 water molecules are included in the QM region. For the remaining potentials, we see the expected behaviour on the basis of treatment of intermolecular interactions (and computational cost) that the rate of convergence is fastest for the semi-empirical DFTB and PM7 methods, followed by TIP3P and QM-only methods. The only exception is the ONIOM(ωB97X-D/6-31G(d):PM6) method which has an exceedingly large error of about 40 kJ mol^−1^ for ΔE(10) in both systems. Another unexpected behaviour is that electronic embedding did not improve the convergence relative to mechanical embedding, even though it includes polarisation of the QM region by the MM subsystem. The first finding is not so surprising since the PM6 method is known to perform poorly for predicting intermolecular interactions, whilst the PM7 method contains corrections for dispersion and hydrogen bonds that are present extensively in the solvated clusters [22]. The poorer performance of ONIOM-EE versus ONIOM-ME models has been reported, but this is likely to be system-dependent as indicated by the studies from Ryde [19] and Ochsenfeld [17], and from this study (vide infra).

Figure 5 shows the convergence profiles for the cationic acids which also lead to some interesting observations. As shown, the QM/EFP method performed the best for the smallest QM region calculations (*m* = 10) with errors 5.0 and 7.5 kJ mol^−1^ for the CH_3_NH_3_^+^ and H-imidazole^+^ systems, respectively. The corresponding errors for the 480-water cluster are 9.0 and 10.0 kJ mol^−1^, respectively. In contrast to the HA/A^−^ systems, the rate of convergence is faster for the TIP3P model followed by PM7 and DFTB and QM-only methods. In these systems, electronic embedding also provided a modest improvement in the convergence compared to mechanical embedding. Another notable difference is that for small QM region sizes (*m* < 50), the errors in the cationic acids appear to be significantly higher compared to the HA/A^−^ systems. For the smallest QM region (*m* = 10), the errors for the worst performing QM-only method are about 169 and 205 kJ mol^−1^ for 160-water clusters of imidazole and methylamine, respectively (compared to ~30–40 kJ mol^−1^ for the HA/A^−^ systems). The larger errors in the cationic systems suggest that water may not shield cations as well as it shields anions. This is supported by experimental gas phase studies of ion-water clusters of monoatomic cations (e.g., Li^+^, Na^+^, and K^+^) which have clustering free energies that are significantly more exergonic compared to the monoatomic anions in the same period (F^−^, Cl^−^, and Br^−^) despite their larger ionic radii [43]. This may also explain the slower rate of convergence for the PM6, PM7, and DFTB potentials in the cationic systems relative to the neutral acids.

The good performance of the TIP3P water potential observed in cationic systems but not the HA/A^−^ systems is particularly interesting. To better understand these results, we refer to the ONIOM reaction scheme in Figure 3, where it becomes clear that differences in the ONIOM models employing different embedding potentials are due to the ΔE^L^(full, *m*) contribution. In particular, the accuracy of the ONIOM models relies on the ability of the embedding potential to reproduce ΔE^L^(full, *m*) relative to the ωB97X-D/6-31G(d) values. Table 1 presents ΔE^L^(full, 10) values of two random snapshots for the CH_3_NH_3_^+^/CH_3_NH_2_ and C_6_H_5_OH/C_6_H_5_O^−^ systems. Formally, ΔE^L^(full, *m*) corresponds to an isodesmic proton exchange reaction and should, therefore, be less sensitive to the choice of electronic structure method due to systematic error cancellation. This is evident from the good agreement between the ωB97X-D and HF values (within 5 kJ mol^−1^) shown in Table 1. Thus, what is really needed is an embedding potential that display similar systematic errors to the QM level of theory for describing the isodesmic proton exchange reaction. Accordingly, the TIP3P model outperformed DFTB and PM7 due to better cancellation of errors for the cationic acids.

The data in Table 1 also indicates that the different combinations of QM and MM may lead to quite different results. For example, because the errors in AMBER/TIP3P are more similar to the PM7 and DFTB semi-empirical methods, the ONIOM(PM7:TIP3P) combination should provide a reasonably accurate approximation to the pure PM7 calculation of the full system. In other words, we would expect the ONIOM(PM7:TIP3P) model to converge faster to the PM7 result for the full system, when compared to the convergence of ONIOM(ωB97X-D/6-31G(d):TIP3P) calculations towards the ωB97X-D/6-31G(d) results for the full system. This may partly explain some of the diverging conclusions reported in the literature. In a recent study on proton transfer in DNA, Major and co-workers observed rapid convergence of energy profiles with QM region size (64 atoms) when using the AM1/d-PhoT/CHARMM model [18], whereas a similar study by Roßbach and Ochsenfeld using B3LYP-D3/AMBER suggests that about 1150 QM atoms are needed for convergence [17]. 

Ultimately, it is important to recognise that QM/MM are approximations to the pure QM calculation of the full system (instead of experiment), so it is interesting to note that a relatively large QM region is needed for these approximations to fall within 10 kJ mol^−1^ of the pure QM result. The results are summarised in Table 2 for the 160-water clusters, and the values in parenthesis refer to the unsigned errors associated with the minimal (*m* = 10) QM region calculations. As shown, the EFP method consistently performs the best for all four systems and its use is recommended when using a small QM region. In terms of the minimal QM region needed for the error in the QM/MM approximation falling below 10 kJ mol^−1^, the semi-empirical PM7 and DFTB methods work best for neutral acids, while the TIP3P (with electronic embedding) is recommended for cationic acids. In this paper, the QM region is radially expanded based on distance from the reaction centre, and the data in Table 2 are probably upper bound values of the minimal QM region needed to obtain this level of accuracy. In the future, it would be interesting to examine the use of systematic QM region determination methods, e.g., charge shift analysis [44], to see if this results in a significant reduction in the size of minimal QM region.

## 4. Summary and Conclusions

In this paper, we set out to address the question of whether the use of more sophisticated embedding potentials lead to better accuracy and faster QM region size convergence of QM/MM models. In context of QM/MM and QM/QM′ modelling of solvated acid clusters, this study presents several interesting results and lessons: (1) The PM6 method is not recommended for use in the ‘low-level’ subsystem especially where there is extensive hydrogen bonding as is the case for water clusters. (2) The EFP method is recommended for QM/MM calculations involving small QM regions. (3) The use of more robust semi-empirical methods or electronic embedding does not necessarily lead to improved performance relative to fixed charge TIP3P potential with mechanical embedding. With respect to the last point, the analysis presented herein indicates that it is the ability of the embedding potential to reproduce ΔE^L^(full, *m*) (Figure 3) relative to the QM values that is critical for the accuracy of the resulting ONIOM model. We showed that the extent of error cancellation is not necessarily better when using semi-empirical potentials compared to fixed charge force fields, and is highly sensitive to the nature of the system (cationic vs. neutral acids). Also, the pairing of a QM with MM (or QM′) method can affect the accuracy of the model relative to the pure QM calculation of the full system. For example, for the purpose of reproducing the corresponding full QM results, the PM7/TIP3P combination is likely to result in much smaller errors compared to HF/TIP3P or ωB97X-D/TIP3P models. In keeping with the findings of this work, it should be emphasised that these conclusions may not hold beyond the systems examined herein, but it confirms that the good performance of a particular QM/MM procedure may not be readily transferable to other systems, and the better quality potentials do not necessarily lead to improved performance. 

## Figures and Tables

**Table 1:** ΔE^L^(full, 10) values determined at various levels of theory for two snapshots. Values in parenthesis refer to relative values.

Theory	CH_3_NH_3_^+^		Phenol	
	Frame 0	Frame 10	Frame 0	Frame 10
ωB97X-D/6-31G(d)	296.0 (0.0)	206.7 (0.0)	−31.2 (0.0)	−46.9 (0.0)
AMBER/TIP3P	258.2 (−37.9)	173.3 (−33.4)	−5.1 (26.2)	−1.3 (45.7)
PM7	240.9 (−55.2)	172.9 (−33.7)	−18.1 (13.2)	−24.8 (22.1)
DFTB	226.5 (−69.5)	156.5 (−50.1)	−18.9 (12.4)	−32.6 (14.3)
HF/6-31G(d)	296.9 (0.9)	204.8 (−1.9)	−28.7 (2.5)	−51.4 (-4.4)

**Table 2:** The minimal quantum mechanics (QM) region size (*m*) needed to approach within 10 kJ mol^−1^ of the pure QM result on the full system (160-water cluster). Values in parenthesis refer to error associated with smallest (*m* = 10) QM region.

Method	HCOOH	C_6_H_5_OH	CH_3_NH_3_^+^	H-Imidazole^+^
QM-only	>150 (28.0)	150 (35.0)	150 (204.9)	150 (168.8)
TIP3P	60 (28.0)	50 (32.9)	60 (32.5)	50 (14.6)
TIP3P-EE	70 (30.3)	70 (32.5)	30 (21.0)	30 (11.0)
EFP	40 (11.7)	10 (8.9)	10 (5.1)	10 (7.5)
PM6	80 (43.1)	100 (39.1)	110 (79.9)	110 (71.0)
PM7	30 (14.9)	40 (17.0)	70 (36.1)	60 (22.3)
DFTB	30 (10.4)	40 (11.4)	70 (45.2)	70 (35.0)

## Supplementary Materials

Supplementary materials are available online. Tables of errors in the QM/MM and QM/QM′ models for the 160- and 480-water clusters. (Tables S1–S8), and an example Gaussian ONIOM input file (Table S9).
