# Supplementary material for: Do Better Quality Embedding Potentials Accelerate the Convergence of QM/MM Models? The Case of Solvated Acid Clusters
Source: Molecules. 2018 Sep 26;23(10):2466. doi: 10.3390/molecules23102466 (PMC6222911; doi:10.3390/molecules23102466)
Supplement: Supplementary file 1 [file molecules-23-02466-s001.pdf]

Supplementary Information

Do Better Quality Embedding Potentials Accelerate the Convergence of QM/MM Models? The Case of Solvated Acid Clusters

Junming Ho [1*], Yihan Shao [2] and Jin Kato [1]

[1] School of Chemistry, University of New South Wales, Sydney, NSW 2052, Australia
[2] Department of Chemistry and Biochemistry, University of Oklahoma, Norman, Oklahoma 73019, United States; yihan.shao@ou.edu
* Correspondence: junming.ho@unsw.edu.au ; Tel.: +612-93856430

CONTENTS

1. Mean absolute errors in QM/MM deprotonation energies for 160-water clusters **S2**

2. Mean absolute errors in QM/MM deprotonation energies for 480-water clusters **S6**

3. Example QM/MM input file for a selected frame for the $C_6H_5OH/C_6H_5O^-$ system **S10**

Table S1. Mean absolute errors of various QM/MM and QM/QM' models (relative to pure ωB97XD/6-31G(d) calculation of the full system) for simulations of the deprotonation energy of $HCOOH(H_2O)_{160}$ (in kJ mol^{-1}). Values in parenthesis refer to standard deviation over 30 snapshots.

n(H_2O)	PM6		PM7		DFTB		EFP		TIP3P		TIP3P-EE		QM only	
0	50.98	(13.55)	26.67	(15.20)	27.49	(14.84)	29.68	(11.92)	71.17	(15.84)	71.85	(15.83)	44.94	(32.51)
10	43.11	(8.90)	14.91	(8.20)	10.44	(8.32)	11.66	(5.36)	28.03	(13.02)	30.31	(10.05)	28.03	(21.54)
20	31.19	(9.08)	12.54	(8.22)	12.03	(6.97)	12.03	(4.58)	18.09	(10.71)	24.23	(10.17)	30.40	(23.33)
30	27.50	(8.31)	9.06	(6.09)	9.57	(6.34)	10.71	(4.16)	12.95	(9.32)	15.67	(10.10)	26.38	(21.38)
40	22.96	(5.32)	6.27	(4.03)	7.66	(4.14)	9.81	(2.97)	10.44	(6.67)	12.55	(7.52)	19.59	(15.76)
50	18.04	(4.81)	5.19	(4.39)	6.42	(4.68)	9.44	(2.39)	10.32	(6.43)	11.66	(6.79)	15.88	(13.14)
60	14.13	(4.37)	5.47	(3.60)	6.42	(3.98)	9.13	(2.16)	9.14	(6.30)	11.31	(5.86)	18.40	(13.86)
70	11.84	(3.64)	4.81	(3.70)	5.43	(3.97)	8.57	(1.84)	7.79	(4.84)	9.29	(4.95)	17.24	(14.21)
80	9.40	(3.58)	5.52	(3.09)	5.75	(3.63)	7.91	(1.95)	7.31	(5.39)	8.06	(3.97)	20.36	(12.46)
90	8.62	(3.17)	4.23	(2.96)	4.41	(3.46)	6.85	(1.77)	5.84	(4.59)	5.67	(3.85)	17.71	(12.42)
100	7.21	(3.68)	4.10	(2.87)	4.19	(3.16)	5.86	(1.85)	5.81	(4.12)	5.21	(3.82)	16.67	(9.23)
110	5.96	(2.97)	3.58	(2.80)	3.73	(2.65)	4.78	(1.56)	4.58	(4.07)	4.17	(3.67)	13.61	(8.86)
120	4.69	(2.61)	3.41	(2.84)	3.55	(2.85)	3.63	(1.16)	3.21	(2.72)	3.26	(2.44)	15.11	(10.30)
130	3.55	(2.12)	2.86	(1.63)	2.98	(1.59)	2.55	(1.02)	2.89	(1.87)	2.86	(1.73)	12.38	(7.78)
140	2.53	(1.68)	2.35	(1.76)	2.46	(1.80)	1.56	(0.80)	2.12	(1.87)	2.12	(1.40)	10.37	(7.62)
150	1.67	(1.32)	2.43	(1.80)	2.61	(1.93)	0.68	(0.53)	1.82	(1.14)	1.62	(1.20)	11.09	(8.22)
160	0.00	(0.00)	0.00	(0.00)	0.00	(0.00)	0.00	(0.00)	0.00	(0.00)	0.00	(0.00)	0.00	(0.00)

Table S2. Mean absolute errors of various QM/MM and QM/QM' models (relative to pure ωB97XD/6-31G(d) calculation of the full system) for simulations of the deprotonation energy of $C_6H_5OH(H_2O)_{160}$ (in kJ mol^{-1}). Values in parenthesis refer to standard deviation over 30 snapshots.

n(H2O)	PM6		PM7		DFTB		EFP		TIP3P		TIP3P-EE		QM only	
0	46.35	(10.65)	23.72	(12.29)	25.73	(10.99)	21.64	(8.53)	57.43	(14.13)	61.33	(13.37)	63.06	(28.18)
10	39.12	(7.43)	16.99	(7.01)	11.40	(7.46)	8.88	(4.24)	32.87	(11.14)	32.51	(11.61)	34.96	(24.87)
20	28.81	(6.94)	15.81	(8.22)	12.74	(6.34)	10.52	(3.42)	22.80	(11.06)	29.30	(9.61)	43.64	(26.84)
30	22.76	(8.40)	13.62	(8.16)	11.67	(8.28)	11.07	(3.57)	17.32	(10.15)	23.56	(9.19)	38.05	(32.33)
40	20.39	(5.85)	8.13	(6.56)	8.17	(5.43)	10.19	(2.93)	11.39	(9.39)	16.36	(8.01)	28.43	(19.35)
50	18.70	(5.88)	6.70	(4.71)	6.54	(4.20)	9.18	(2.46)	9.53	(7.76)	12.18	(7.48)	25.32	(15.52)
60	16.01	(6.04)	6.48	(4.90)	6.08	(4.08)	8.34	(2.37)	9.39	(7.58)	10.38	(7.80)	22.38	(15.78)
70	14.16	(5.20)	5.89	(4.29)	5.45	(4.17)	7.72	(2.07)	8.15	(5.89)	8.05	(5.71)	22.44	(16.04)
80	11.64	(5.34)	6.25	(5.06)	5.75	(4.96)	7.02	(1.92)	7.96	(6.23)	7.17	(5.61)	23.82	(17.50)
90	10.22	(5.53)	6.87	(4.74)	6.28	(4.99)	6.14	(1.70)	7.92	(5.32)	5.77	(5.20)	24.61	(17.09)
100	8.18	(4.92)	5.81	(3.95)	5.75	(3.69)	5.27	(1.68)	6.36	(4.86)	4.59	(3.95)	20.72	(14.86)
110	7.00	(4.85)	5.66	(4.56)	5.53	(4.13)	4.20	(1.55)	5.40	(4.04)	4.52	(3.24)	21.95	(15.98)
120	5.52	(3.66)	4.65	(3.84)	4.49	(3.71)	3.38	(1.26)	4.33	(2.51)	3.26	(2.36)	18.92	(15.83)
130	4.45	(2.74)	4.38	(3.93)	4.47	(3.65)	2.58	(1.04)	3.59	(2.67)	3.22	(2.65)	18.05	(15.26)
140	2.89	(1.90)	3.14	(2.48)	2.99	(2.30)	1.80	(0.90)	2.47	(2.46)	2.40	(2.12)	11.87	(10.42)
150	1.67	(1.18)	2.13	(1.78)	2.12	(1.80)	0.86	(0.49)	1.50	(1.31)	1.52	(1.28)	9.03	(7.31)
160	0.00	(0.00)	0.00	(0.00)	0.00	(0.00)	0.00	(0.00)	0.00	(0.00)	0.00	(0.00)	0.00	(0.00)

Table S3. Mean absolute errors of various QM/MM and QM/QM' models (relative to pure ωB97XD/6-31G(d) calculation of the full system) for simulations of the deprotonation energy of $CH_3NH_3^+(H_2O)_{160}$ (in kJ mol^{-1}). Values in parenthesis refer to standard deviation over 30 snapshots.

n(H2O)	PM6		PM7		DFTB		EFP		TIP3P		TIP3P-EE		QM only	
0	188.37	(12.92)	121.73	(14.69)	123.09	(12.36)	9.57	(6.76)	74.11	(19.03)	58.83	(17.60)	502.71	(45.39)
10	79.93	(9.10)	36.06	(9.57)	45.24	(10.97)	5.14	(4.00)	32.50	(14.40)	21.02	(11.22)	204.87	(38.87)
20	60.30	(8.05)	29.06	(9.39)	31.17	(8.84)	6.40	(4.17)	27.64	(13.96)	18.12	(10.23)	151.09	(36.49)
30	41.27	(6.96)	16.33	(8.71)	16.95	(8.99)	8.72	(3.67)	15.81	(8.33)	9.09	(5.98)	91.10	(27.27)
40	35.08	(5.65)	15.59	(7.68)	15.93	(7.96)	7.70	(2.99)	12.75	(8.08)	7.66	(5.44)	82.40	(29.64)
50	30.33	(4.36)	15.18	(6.44)	15.04	(6.48)	7.22	(2.53)	10.91	(7.10)	7.96	(5.61)	76.15	(28.02)
60	24.48	(4.35)	11.40	(5.11)	11.48	(4.97)	7.35	(2.32)	9.21	(6.62)	6.28	(5.21)	58.70	(20.13)
70	19.45	(4.43)	8.55	(5.43)	8.80	(5.35)	7.76	(2.21)	7.80	(5.52)	6.40	(4.54)	46.24	(22.37)
80	15.63	(4.11)	6.62	(4.24)	6.60	(4.26)	7.22	(2.11)	6.83	(5.01)	5.10	(3.62)	32.95	(19.60)
90	12.91	(3.34)	5.40	(3.72)	5.25	(3.31)	6.41	(1.59)	5.54	(4.51)	3.68	(3.24)	28.16	(14.37)
100	11.15	(2.94)	5.53	(3.17)	5.16	(3.33)	5.41	(1.47)	4.73	(3.51)	3.84	(2.58)	26.17	(17.66)
110	9.14	(2.24)	4.60	(3.45)	4.32	(3.33)	4.52	(1.36)	3.93	(3.47)	2.74	(2.29)	24.16	(17.24)
120	7.49	(2.37)	4.04	(3.35)	4.09	(3.20)	3.50	(1.22)	3.96	(2.70)	3.20	(2.26)	19.93	(14.72)
130	5.47	(2.26)	4.07	(2.40)	3.89	(2.36)	2.64	(1.04)	3.49	(2.26)	2.66	(1.97)	18.77	(10.37)
140	3.64	(1.82)	3.03	(1.93)	2.99	(1.90)	1.65	(0.91)	2.41	(1.90)	1.96	(1.59)	13.39	(9.95)
150	1.80	(1.29)	2.00	(1.48)	1.89	(1.42)	0.80	(0.52)	1.69	(1.37)	1.35	(1.21)	9.24	(5.74)
160	0.00	(0.00)	0.00	(0.00)	0.00	(0.00)	0.00	(0.00)	0.00	(0.00)	0.00	(0.00)	0.00	(0.00)

Table S4. Mean absolute errors of various QM/MM and QM/QM' models (relative to pure ωB97XD/6-31G(d) calculation of the full system) for simulations of the deprotonation energy of H^+Imidazole$(H_2O)_{160}$ (in kJ mol^{-1}). Values in parenthesis refer to standard deviation over 30 snapshots.

n(H2O)	PM6		PM7		DFTB		EFP		TIP3P		TIP3P-EE		QM only	
0	152.36	(10.14)	88.83	(12.10)	106.42	(9.34)	12.29	(11.55)	38.42	(14.46)	31.81	(14.37)	380.28	(36.57)
10	70.96	(9.89)	22.29	(10.30)	34.99	(10.96)	7.47	(5.06)	14.55	(10.35)	10.97	(6.97)	168.76	(40.17)
20	55.42	(7.78)	20.48	(8.46)	27.39	(8.68)	7.95	(4.30)	18.01	(12.08)	10.75	(6.68)	126.49	(29.87)
30	40.47	(8.25)	13.69	(9.38)	16.98	(10.71)	9.16	(3.91)	13.06	(9.38)	8.94	(5.61)	83.87	(33.71)
40	33.66	(5.69)	12.46	(6.66)	14.00	(6.49)	8.28	(3.49)	11.51	(8.26)	6.57	(5.59)	73.78	(26.24)
50	28.01	(5.16)	11.17	(6.61)	12.01	(6.19)	7.83	(2.91)	9.98	(6.28)	6.53	(5.12)	63.86	(23.52)
60	23.38	(5.50)	9.79	(6.71)	10.28	(6.33)	7.45	(2.70)	8.78	(5.64)	5.97	(5.08)	51.94	(27.05)
70	19.21	(5.11)	7.94	(4.79)	8.08	(4.69)	7.24	(2.50)	8.09	(5.81)	4.56	(4.06)	38.67	(20.19)
80	16.42	(5.39)	7.10	(5.23)	6.79	(4.93)	6.89	(2.51)	7.68	(6.39)	5.79	(3.71)	32.46	(19.93)
90	14.19	(4.61)	7.25	(4.12)	7.52	(3.57)	5.85	(2.06)	6.62	(5.27)	4.88	(3.64)	33.30	(16.10)
100	11.04	(4.42)	5.90	(4.30)	5.81	(4.49)	5.17	(1.66)	5.43	(4.03)	4.43	(3.13)	25.39	(15.98)
110	8.39	(3.84)	4.68	(3.45)	4.47	(3.24)	4.30	(1.70)	4.14	(3.99)	3.97	(3.15)	20.18	(13.74)
120	6.21	(3.23)	4.15	(3.58)	4.01	(3.42)	3.42	(1.27)	3.39	(3.29)	3.43	(2.82)	18.59	(13.04)
130	4.22	(2.69)	3.28	(2.52)	3.33	(2.38)	2.78	(0.86)	2.78	(2.99)	2.76	(2.43)	13.75	(8.78)
140	2.83	(1.94)	2.43	(1.60)	2.37	(1.55)	1.64	(0.80)	2.77	(2.12)	2.09	(1.69)	10.51	(6.12)
150	1.70	(1.44)	2.19	(1.47)	2.24	(1.44)	0.69	(0.42)	1.85	(1.40)	1.55	(1.25)	8.69	(7.07)
160	0.00	(0.00)	0.00	(0.00)	0.00	(0.00)	0.00	(0.00)	0.00	(0.00)	0.00	(0.00)	0.00	(0.00)

Table S5. Mean absolute errors of various QM/MM and QM/QM' models (relative to pure ωB97XD/6-31G(d) calculation of the full system) for simulations of the deprotonation energy of $HCOOH(H_2O)_{480}$ (in kJ mol^{-1}). Values in parenthesis refer to standard deviation over 30 snapshots.

n(H2O)	PM6		PM7		DFTB		EFP		TIP3P		TIP3P-EE		QM only	
30	46.40	(8.70)	9.88	(7.67)	11.45	(7.50)	11.31	(4.84)	16.62	(11.37)	21.03	(11.38)	26.65	(24.33)
60	32.65	(5.13)	7.55	(4.91)	9.52	(6.28)	10.29	(2.71)	14.24	(7.31)	17.58	(7.27)	24.38	(16.83)
90	27.15	(4.16)	5.50	(4.86)	6.80	(5.36)	9.34	(2.25)	10.72	(6.10)	13.25	(6.73)	18.88	(15.00)
120	23.12	(4.34)	5.21	(4.47)	6.18	(4.75)	8.71	(2.12)	7.81	(6.00)	10.80	(6.14)	22.28	(14.13)
150	19.83	(3.59)	4.52	(3.48)	5.38	(4.04)	8.72	(1.88)	6.60	(5.63)	8.70	(5.56)	19.63	(14.44)
180	16.02	(3.26)	4.07	(3.04)	4.30	(3.41)	8.97	(1.84)	6.93	(4.99)	8.65	(5.02)	16.58	(10.38)
210	13.63	(3.21)	3.84	(2.75)	4.11	(3.08)	8.58	(1.40)	6.24	(3.80)	7.64	(4.39)	14.60	(11.23)
240	11.83	(3.36)	3.74	(2.53)	3.75	(2.75)	8.51	(1.38)	5.30	(3.52)	6.51	(3.99)	15.01	(8.90)
270	9.91	(2.86)	3.36	(2.54)	3.48	(2.66)	8.06	(1.19)	4.29	(3.33)	5.38	(3.51)	14.19	(10.01)
300	7.68	(2.55)	2.93	(2.69)	3.23	(2.58)	7.63	(1.01)	3.87	(2.90)	4.46	(2.93)	12.17	(9.23)
330	6.38	(2.21)	2.99	(2.38)	3.33	(2.68)	6.66	(0.94)	3.11	(2.51)	3.20	(2.25)	12.66	(8.77)
360	5.25	(1.96)	2.55	(2.69)	2.65	(2.94)	5.33	(0.97)	2.37	(2.43)	2.27	(2.11)	9.71	(11.00)
390	3.44	(2.00)	2.92	(2.21)	3.19	(2.44)	4.22	(0.98)	2.49	(1.83)	2.21	(1.71)	12.26	(9.65)
420	2.31	(1.39)	3.05	(1.89)	3.28	(2.04)	3.24	(0.73)	2.27	(1.55)	2.02	(1.57)	13.77	(8.40)
450	1.55	(1.05)	2.62	(1.64)	2.64	(1.58)	1.92	(0.58)	1.61	(1.00)	1.81	(1.25)	11.78	(7.30)
480	0.00	(0.00)	0.00	(0.00)	0.00	(0.00)	0.00	(0.00)	0.00	(0.00)	0.00	(0.00)	0.00	(0.00)

Table S6. Mean absolute errors of various QM/MM and QM/QM' models (relative to pure ωB97XD/6-31G(d) calculation of the full system) for simulations of the deprotonation energy of $C_6H_5OH(H_2O)_{480}$ (in kJ mol^{-1}). Values in parenthesis refer to standard deviation over 30 snapshots.

n(H2O)	PM6		PM7		DFTB		EFP		TIP3P		TIP3P-EE		QM only	
30	41.59	(7.64)	14.53	(7.04)	13.81	(7.29)	11.66	(4.18)	23.63	(10.87)	30.52	(10.01)	37.46	(27.15)
60	34.83	(6.00)	6.08	(5.29)	6.66	(4.58)	9.29	(2.86)	14.64	(10.13)	16.90	(9.06)	17.72	(13.77)
90	28.78	(5.37)	5.57	(4.31)	6.33	(4.25)	8.50	(2.71)	11.80	(7.67)	13.23	(8.09)	17.29	(12.66)
120	24.06	(4.22)	4.58	(3.26)	5.26	(2.98)	8.43	(2.39)	8.90	(6.37)	10.74	(6.62)	15.31	(11.85)
150	19.82	(3.26)	3.98	(2.80)	4.47	(3.02)	8.70	(1.95)	8.11	(5.29)	9.71	(5.07)	15.71	(9.53)
180	16.68	(3.60)	3.87	(3.05)	4.59	(2.86)	8.48	(1.91)	6.81	(4.45)	8.53	(5.03)	13.16	(11.51)
210	13.79	(3.14)	3.61	(3.27)	4.04	(3.11)	8.44	(1.54)	5.60	(4.17)	8.10	(4.59)	13.55	(14.03)
240	12.55	(3.25)	3.13	(2.90)	3.28	(2.65)	7.91	(1.35)	4.64	(3.49)	6.30	(3.88)	10.03	(11.09)
270	10.34	(2.64)	2.83	(1.88)	2.84	(2.15)	7.73	(1.44)	3.83	(3.35)	4.47	(3.37)	8.78	(6.69)
300	8.27	(2.39)	3.04	(1.73)	3.05	(1.84)	7.54	(1.04)	3.40	(3.17)	3.74	(2.58)	11.31	(7.06)
330	6.66	(2.31)	3.02	(1.81)	2.95	(1.86)	7.09	(1.05)	3.19	(2.44)	2.92	(2.09)	10.98	(8.93)
360	5.19	(2.36)	3.01	(2.23)	3.15	(2.32)	5.84	(1.26)	2.74	(2.21)	2.39	(1.91)	12.30	(8.90)
390	3.54	(1.73)	2.11	(1.80)	2.29	(1.82)	4.45	(0.93)	2.29	(1.43)	1.98	(1.44)	9.31	(7.24)
420	2.62	(1.32)	1.77	(1.41)	1.83	(1.50)	3.00	(1.04)	1.82	(1.33)	1.26	(1.21)	7.96	(5.14)
450	1.45	(0.86)	1.51	(0.99)	1.47	(1.12)	1.63	(0.57)	1.18	(0.98)	1.05	(0.82)	6.92	(4.50)
480	0.00	(0.00)	0.00	(0.00)	0.00	(0.00)	0.00	(0.00)	0.00	(0.00)	0.00	(0.00)	0.00	(0.00)

Table S7. Mean absolute errors of various QM/MM and QM/QM' models (relative to pure ωB97XD/6-31G(d) calculation of the full system) for simulations of the deprotonation energy of $CH_3NH_3^+(H_2O)_{480}$ (in kJ mol^{-1}). Values in parenthesis refer to standard deviation over 30 snapshots.

n(H2O)	PM6		PM7		DFTB		EFP		TIP3P		TIP3P-EE		QM only	
30	63.72	(7.36)	21.24	(9.70)	22.47	(10.42)	9.02	(3.97)	19.52	(10.43)	11.49	(8.13)	119.15	(34.20)
60	46.92	(4.64)	16.30	(6.07)	17.15	(6.68)	8.03	(2.90)	13.41	(8.73)	9.50	(7.56)	85.82	(26.75)
90	35.35	(3.71)	9.80	(4.56)	10.03	(5.20)	8.47	(2.21)	9.61	(7.39)	6.94	(6.12)	52.81	(25.51)
120	29.93	(3.24)	8.85	(3.87)	9.48	(4.20)	8.20	(2.05)	8.72	(5.76)	6.81	(5.32)	45.78	(19.52)
150	24.02	(2.68)	6.38	(2.77)	7.10	(3.10)	8.12	(1.94)	6.59	(4.42)	4.47	(3.91)	32.37	(16.44)
180	20.20	(2.62)	5.14	(3.54)	5.64	(3.84)	8.24	(2.11)	6.52	(4.40)	4.50	(4.00)	27.87	(17.69)
210	16.40	(2.76)	4.44	(2.71)	4.56	(2.96)	8.12	(1.83)	5.35	(3.79)	3.67	(3.07)	24.48	(13.24)
240	13.89	(2.49)	4.00	(2.63)	4.28	(3.02)	8.01	(1.58)	4.62	(2.67)	4.00	(2.18)	20.71	(14.52)
270	11.54	(2.06)	3.12	(1.89)	3.52	(1.88)	7.77	(1.54)	3.76	(3.15)	3.32	(2.66)	16.18	(9.55)
300	9.66	(2.19)	3.13	(2.39)	3.15	(2.33)	7.22	(1.24)	3.79	(3.42)	3.16	(2.80)	15.69	(10.74)
330	7.48	(2.18)	2.97	(1.98)	2.75	(2.16)	6.47	(1.19)	3.52	(2.66)	2.93	(2.04)	13.97	(10.79)
360	5.85	(1.87)	2.66	(1.93)	2.53	(1.99)	5.12	(1.04)	2.73	(2.38)	2.30	(1.63)	11.42	(9.20)
390	4.21	(1.73)	2.26	(1.96)	2.33	(1.97)	3.86	(0.87)	2.37	(1.94)	2.08	(1.48)	10.26	(9.03)
420	2.75	(1.58)	2.32	(1.82)	2.24	(1.83)	2.43	(0.76)	1.71	(1.37)	1.63	(1.23)	9.87	(7.85)
450	1.23	(0.83)	1.57	(1.29)	1.63	(1.28)	1.26	(0.52)	0.94	(0.73)	0.99	(0.75)	7.05	(5.17)
480	0.00	(0.00)	0.00	(0.00)	0.00	(0.00)	0.00	(0.00)	0.00	(0.00)	0.00	(0.00)	0.00	(0.00)

Table S8. Mean absolute errors of various QM/MM and QM/QM' models (relative to pure ωB97XD/6-31G(d) calculation of the full system) for simulations of the deprotonation energy of H^+Imidazole$(H_2O)_{480}$ (in kJ mol^{-1}). Values in parenthesis refer to standard deviation over 30 snapshots.

n(H2O)	PM6		PM7		DFTB		EFP		TIP3P		TIP3P-EE		QM only	
30	64.51	(8.34)	21.27	(9.96)	25.16	(10.23)	9.99	(4.72)	16.53	(12.56)	11.59	(7.70)	129.75	(36.85)
60	47.42	(6.60)	17.15	(9.00)	17.64	(8.74)	8.74	(3.68)	11.32	(8.83)	9.30	(7.08)	97.82	(30.58)
90	38.23	(5.20)	13.86	(6.69)	14.28	(5.90)	8.80	(2.83)	10.62	(6.32)	7.92	(4.30)	76.52	(22.73)
120	30.06	(4.70)	9.48	(5.74)	9.65	(5.98)	8.89	(2.28)	8.16	(3.99)	5.34	(3.28)	54.65	(19.77)
150	25.57	(4.39)	8.80	(5.33)	8.67	(4.90)	8.61	(2.00)	7.46	(4.46)	5.11	(4.09)	48.26	(19.45)
180	21.11	(3.94)	6.91	(4.71)	7.20	(4.24)	8.53	(1.75)	6.19	(3.83)	4.99	(2.95)	38.69	(16.85)
210	17.20	(3.16)	5.18	(3.86)	5.48	(4.04)	8.59	(1.57)	4.56	(2.85)	3.30	(2.36)	29.78	(14.94)
240	14.19	(3.33)	4.13	(3.10)	4.19	(2.65)	8.36	(1.71)	4.85	(3.40)	3.49	(2.89)	21.98	(12.10)
270	11.95	(2.72)	3.65	(2.62)	4.20	(2.28)	8.09	(1.54)	4.30	(2.59)	3.28	(2.46)	20.56	(10.93)
300	10.12	(3.09)	4.12	(3.38)	4.25	(3.12)	7.26	(1.36)	3.95	(2.52)	3.19	(2.17)	18.47	(13.85)
330	8.38	(2.28)	3.61	(3.06)	3.73	(3.05)	6.29	(1.10)	2.90	(1.79)	2.58	(1.65)	17.24	(13.77)
360	6.22	(1.82)	2.79	(2.15)	2.52	(2.23)	5.45	(0.85)	2.08	(2.08)	1.68	(1.37)	12.81	(10.79)
390	4.55	(1.57)	2.14	(1.67)	1.94	(1.39)	4.34	(0.80)	2.17	(1.47)	1.53	(1.24)	10.33	(7.46)
420	2.89	(1.48)	2.02	(1.40)	1.96	(0.85)	3.01	(0.57)	1.55	(1.29)	1.28	(1.01)	9.14	(6.99)
450	1.68	(1.18)	1.74	(1.47)	1.69	(1.34)	1.82	(0.63)	1.21	(1.03)	1.16	(1.05)	7.62	(6.47)
480	0.00	(0.00)	0.00	(0.00)	0.00	(0.00)	0.00	(0.00)	0.00	(0.00)	0.00	(0.00)	0.00	(0.00)

Table S9. Example Gaussian ONIOM input file

Phenol	Phenoxide
E(ωB97XD/6-31G(d)) = -12532.01435040 H	E(ωB97XD/6-31G(d)) = -12531.4331048000 H
E[ONIOM(ωB97XD/6-31G(d):PM7)] = -1086.111020840625 H	E[ONIOM(ωB97XD/6-31G(d):PM7)] -1085.524766389580 H
%mem=8gb	%mem=8gb
%nproc=8	%nproc=8
#ONIOM(wB97XD/6-31G(d):PM7)	#ONIOM(wB97XD/6-31G(d):PM7)
ONIOM SP 10 water in QM	ONIOM SP 10 water in QM
0 1 0 1	-1 1 -1 1
C -1 -4.285746 1.066096 -0.521942 H	C -1 -4.285746 1.066096 -0.521942 H
C -1 -4.688949 2.320446 -0.068269 H	C -1 -4.688949 2.320446 -0.068269 H
C -1 -5.144488 2.435399 1.238186 H	C -1 -5.144488 2.435399 1.238186 H
C -1 -5.224815 1.253296 2.051174 H	C -1 -5.224815 1.253296 2.051174 H
C -1 -4.840904 -0.014621 1.555555 H	C -1 -4.840904 -0.014621 1.555555 H
C -1 -4.434834 -0.059305 0.238734 H	C -1 -4.434834 -0.059305 0.238734 H
O 0 -3.805604 0.875467 -1.786253 H	O 0 -3.805604 0.875467 -1.786253 H
H 0 -4.593932 3.154735 -0.695924 H	H 0 -4.593932 3.154735 -0.695924 H
H 0 -5.535233 3.419906 1.546617 H	H 0 -5.535233 3.419906 1.546617 H
H 0 -5.682077 1.342306 3.036961 H	H 0 -5.682077 1.342306 3.036961 H
H 0 -4.888500 -0.901659 2.184003 H	H 0 -4.888500 -0.901659 2.184003 H
H 0 -4.028756 -1.016087 -0.081303 H	H 0 -4.028756 -1.016087 -0.081303 H
H 0 -3.640706 -0.090116 -2.016728 H	O 0 -2.657447 -1.668051 -2.119157 H
O 0 -2.657447 -1.668051 -2.119157 H	H 0 -1.969842 -1.673852 -2.785038 H
H 0 -1.969842 -1.673852 -2.785038 H	H 0 -2.308420 -2.206744 -1.409070 H
H 0 -2.308420 -2.206744 -1.409070 H	O 0 -5.897951 2.291192 -3.129913 H
O 0 -5.897951 2.291192 -3.129913 H	H 0 -6.663455 1.748493 -3.318894 H
H 0 -6.663455 1.748493 -3.318894 H	H 0 -5.333551 1.732339 -2.595715 H
H 0 -5.333551 1.732339 -2.595715 H	O 0 -1.754234 2.867129 -1.878143 H
O 0 -1.754234 2.867129 -1.878143 H	H 0 -2.427501 2.285905 -2.231868 H
H 0 -2.427501 2.285905 -2.231868 H	H 0 -1.532984 2.488313 -1.027392 H
H 0 -1.532984 2.488313 -1.027392 H	O 0 -3.946526 5.623685 1.602390 H
O 0 -3.946526 5.623685 1.602390 H	H 0 -3.340109 5.678647 0.863832 H
H 0 -3.340109 5.678647 0.863832 H	H 0 -4.801161 5.468633 1.200159 H
H 0 -4.801161 5.468633 1.200159 H	O 0 -2.730156 2.982899 3.049569 H
O 0 -2.730156 2.982899 3.049569 H	H 0 -3.570844 3.379872 2.821781 H
H 0 -3.570844 3.379872 2.821781 H	H 0 -2.359308 3.568423 3.709770 H
H 0 -2.359308 3.568423 3.709770 H	O 0 -3.368768 0.401087 4.261813 H
O 0 -3.368768 0.401087 4.261813 H	H 0 -2.583005 -0.079911 4.002105 H
H 0 -2.583005 -0.079911 4.002105 H	H 0 -3.378408 1.170399 3.692341 H
H 0 -3.378408 1.170399 3.692341 H	O 0 -6.515965 5.327818 0.373175 H
O 0 -6.515965 5.327818 0.373175 H	H 0 -6.999121 4.854748 -0.304318 H
H 0 -6.999121 4.854748 -0.304318 H	H 0 -6.568474 6.246178 0.108431 H
H 0 -6.568474 6.246178 0.108431 H	O 0 -8.643856 3.166433 1.338647 H
O 0 -8.643856 3.166433 1.338647 H	H 0 -7.975038 3.836131 1.481546 H
H 0 -7.975038 3.836131 1.481546 H	H 0 -8.742461 2.740228 2.190034 H
H 0 -8.742461 2.740228 2.190034 H	O 0 -4.833367 3.087718 4.929371 H
O 0 -4.833367 3.087718 4.929371 H	H 0 -4.845237 2.176986 5.223751 H
H 0 -4.845237 2.176986 5.223751 H	H 0 -3.912947 3.345099 4.982413 H
H 0 -3.912947 3.345099 4.982413 H	O 0 -5.614870 -2.227881 -2.257185 H
O 0 -5.614870 -2.227881 -2.257185 H	H 0 -4.880960 -2.638962 -1.800435 H
H 0 -4.880960 -2.638962 -1.800435 H	H 0 -5.777592 -2.796123 -3.010082 H
H 0 -5.777592 -2.796123 -3.010082 H	O -1 -5.449800 0.466977 5.859749 L
O -1 -5.449800 0.466977 5.859749 L	H -1 -4.982203 0.528417 6.692702 L
H -1 -4.982203 0.528417 6.692702 L	H -1 -4.758926 0.388133 5.201943 L
H -1 -4.758926 0.388133 5.201943 L	O -1 -7.011806 4.070925 3.663779 L
O -1 -7.011806 4.070925 3.663779 L	H -1 -6.260200 3.863964 4.219195 L
H -1 -6.260200 3.863964 4.219195 L	H -1 -7.704871 3.486087 3.970141 L
H -1 -7.704871 3.486087 3.970141 L	O -1 -7.676451 -0.858135 4.311931 L
O -1 -7.676451 -0.858135 4.311931 L	H -1 -6.910227 -0.398179 4.654821 L
H -1 -6.910227 -0.398179 4.654821 L	H -1 -7.323700 -1.449582 3.647108 L
H -1 -7.323700 -1.449582 3.647108 L	O -1 -7.941441 0.968061 -0.249497 L
O -1 -7.941441 0.968061 -0.249497 L	H -1 -7.191887 0.586426 0.207402 L
H -1 -7.191887 0.586426 0.207402 L	H -1 -7.919888 1.895356 -0.013083 L
H -1 -7.919888 1.895356 -0.013083 L	O -1 -2.047162 5.110317 -0.308714 L
O -1 -2.047162 5.110317 -0.308714 L	H -1 -1.151105 5.258089 -0.006261 L
H -1 -1.151105 5.258089 -0.006261 L	H -1 -2.014495 4.261749 -0.750411 L
H -1 -2.014495 4.261749 -0.750411 L	O -1 -6.697639 -3.307610 2.634462 L
O -1 -6.697639 -3.307610 2.634462 L	H -1 -7.360805 -3.260689 1.945811 L
H -1 -7.360805 -3.260689 1.945811 L	H -1 -5.979123 -3.803774 2.242304 L
H -1 -5.979123 -3.803774 2.242304 L	O -1 -8.619490 1.920148 4.087404 L
O -1 -8.619490 1.920148 4.087404 L	H -1 -8.576596 0.966149 4.022006 L
H -1 -8.576596 0.966149 4.022006 L	H -1 -9.168756 2.083688 4.854081 L
H -1 -9.168756 2.083688 4.854081 L	O -1 -4.065450 4.111758 -4.256964 L
O -1 -4.065450 4.111758 -4.256964 L	H -1 -4.872233 3.714562 -3.928988 L
H -1 -4.872233 3.714562 -3.928988 L	H -1 -3.690797 3.449530 -4.837757 L
H -1 -3.690797 3.449530 -4.837757 L	O -1 -6.009713 6.213374 -2.610685 L
O -1 -6.009713 6.213374 -2.610685 L	H -1 -6.044300 7.123773 -2.317070 L
H -1 -6.044300 7.123773 -2.317070 L	H -1 -5.097225 5.954310 -2.482299 L
H -1 -5.097225 5.954310 -2.482299 L	O -1 -4.539106 -3.981438 4.949656 L
O -1 -4.539106 -3.981438 4.949656 L	H -1 -4.268024 -3.633281 4.100225 L
H -1 -4.268024 -3.633281 4.100225 L	H -1 -5.430403 -3.653792 5.069941 L
H -1 -5.430403 -3.653792 5.069941 L	O -1 -0.897962 2.420090 1.118832 L
O -1 -0.897962 2.420090 1.118832 L	H -1 -0.314094 1.688447 1.318903 L
H -1 -0.314094 1.688447 1.318903 L	H -1 -1.579982 2.373001 1.788805 L
H -1 -1.579982 2.373001 1.788805 L	O -1 -2.304624 -3.311450 3.165410 L
O -1 -2.304624 -3.311450 3.165410 L	H -1 -1.753710 -2.628301 3.547551 L
H -1 -1.753710 -2.628301 3.547551 L	H -1 -1.693704 -3.874380 2.689892 L
H -1 -1.693704 -3.874380 2.689892 L	O -1 -4.539390 -4.622493 1.457741 L

O -1	-4.539390	-4.622493	1.457741	L
H -1	-4.636452	-5.431989	0.956216	L
H -1	-3.614625	-4.392963	1.366324	L
O -1	-2.882038	6.256486	-2.739281	L
H -1	-3.069097	5.452458	-3.223822	L
H -1	-2.458972	5.958483	-1.934022	L
O -1	-1.569279	-1.538289	-4.773978	L
H -1	-1.544139	-0.643426	-5.112831	L
H -1	-0.985153	-2.029999	-5.351256	L
O -1	-8.415366	4.377892	-2.330080	L
H -1	-9.017674	3.684398	-2.599380	L
H -1	-7.591140	4.162834	-2.766697	L
O -1	-2.318294	-4.190829	-3.986645	L
H -1	-2.620136	-4.467699	-4.851784	L
H -1	-2.427393	-3.239868	-3.987391	L
O -1	-6.193246	3.776473	7.205814	L
H -1	-5.861485	3.148090	6.564486	L
H -1	-6.442369	3.241411	7.959391	L
O -1	-8.055896	-0.009292	-2.773200	L
H -1	-8.739741	0.623700	-2.992079	L
H -1	-7.932513	0.087606	-1.828944	L
O -1	-1.447669	-4.028710	-0.670458	L
H -1	-1.018192	-4.872680	-0.530831	L
H -1	-0.734549	-3.430620	-0.894018	L
O -1	-8.972666	6.155608	3.894933	L
H -1	-8.219962	5.585680	3.737290	L
H -1	-9.208751	6.486568	3.028353	L
O -1	-6.837485	-0.642618	-5.435489	L
H -1	-6.893433	-0.647884	-4.479940	L
H -1	-6.663732	-1.554045	-5.670741	L
O -1	-3.327041	6.964484	3.823910	L
H -1	-3.423313	6.715297	2.904742	L
H -1	-2.895402	7.818417	3.797117	L
O -1	0.317635	-2.066230	-1.001831	L
H -1	1.270929	-2.068264	-1.088184	L
H -1	0.147863	-1.528312	-0.228492	L
O -1	-3.738760	-5.551006	-1.995051	L
H -1	-3.235442	-5.416952	-2.798127	L
H -1	-3.285363	-5.024210	-1.336910	L
O -1	-8.104014	-3.132568	0.315510	L
H -1	-8.206142	-2.697946	-0.531192	L
H -1	-8.591403	-3.951434	0.225272	L
O -1	-1.968881	0.853791	-5.989144	L
H -1	-1.049482	0.945796	-6.239089	L
H -1	-2.451677	0.952134	-6.809795	L
O -1	-5.916091	8.266763	2.717082	L
H -1	-5.555069	7.549645	3.238270	L
H -1	-6.258382	8.884418	3.363279	L
O -1	-5.534505	6.150675	5.914353	L
H -1	-5.758186	5.356786	6.400089	L
H -1	-4.912899	5.857303	5.248191	L
O -1	0.122445	3.100332	-3.776031	L
H -1	-0.279009	3.418484	-2.967424	L
H -1	-0.618049	2.887367	-4.343964	L
O -1	-3.918904	-1.918653	-6.776871	L
H -1	-4.552208	-1.975727	-6.061399	L
H -1	-3.149592	-1.506217	-6.384077	L
O -1	-3.362641	-2.303536	6.736642	L
H -1	-3.572252	-2.931952	6.045709	L
H -1	-4.063567	-1.653310	6.690364	L
O -1	-7.954826	0.147587	7.304074	L
H -1	-7.036264	-0.079441	7.159382	L
H -1	-8.428867	-0.673108	7.169998	L
O -1	-7.346237	-4.548746	5.102293	L
H -1	-7.377205	-4.142314	4.236217	L
H -1	-7.859124	-5.351553	5.009128	L
O -1	-5.957361	8.125005	-0.086106	L
H -1	-6.000675	8.011930	0.863404	L
H -1	-6.495277	8.897292	-0.260605	L
O -1	-10.386962	0.222064	1.088626	L
H -1	-10.494214	-0.487988	1.721520	L
H -1	-9.539125	0.051749	0.678263	L
O -1	-3.230219	8.222615	-0.702757	L
H -1	-3.001120	7.610281	-1.401894	L
H -1	-3.986091	7.823297	-0.272137	L
O -1	-8.862668	6.844899	-1.149467	L
H -1	-8.337737	6.172569	-1.583808	L
H -1	-9.375205	7.241492	-1.853919	L
O -1	-10.337684	-1.191895	5.016613	L
H -1	-9.383146	-1.223650	4.952729	L
H -1	-10.635665	-1.971212	4.547462	L
O -1	-2.429477	4.922967	5.229390	L
H -1	-1.513370	4.740396	5.438309	L
H -1	-2.426450	5.822753	4.902879	L
O -1	-6.206245	-4.996752	-3.059023	L
H -1	-5.510215	-5.266240	-2.459731	L
H -1	-6.968934	-5.506525	-2.785766	L
O -1	-10.777814	4.006355	-0.841995	L
H -1	-10.337802	3.455511	-0.194545	L
H -1	-10.065722	4.445111	-1.307451	L
O -1	-10.192779	-2.478376	2.363255	L
H -1	-11.098862	-2.422656	2.059709	L
H -1	-9.678657	-2.593963	1.564162	L

H -1	-4.636452	-5.431989	0.956216	L
H -1	-3.614625	-4.392963	1.366324	L
O -1	-2.882038	6.256486	-2.739281	L
H -1	-3.069097	5.452458	-3.223822	L
H -1	-2.458972	5.958483	-1.934022	L
O -1	-1.569279	-1.538289	-4.773978	L
H -1	-1.544139	-0.643426	-5.112831	L
H -1	-0.985153	-2.029999	-5.351256	L
O -1	-8.415366	4.377892	-2.330080	L
H -1	-9.017674	3.684398	-2.599380	L
H -1	-7.591140	4.162834	-2.766697	L
O -1	-2.318294	-4.190829	-3.986645	L
H -1	-2.620136	-4.467699	-4.851784	L
H -1	-2.427393	-3.239868	-3.987391	L
O -1	-6.193246	3.776473	7.205814	L
H -1	-5.861485	3.148090	6.564486	L
H -1	-6.442369	3.241411	7.959391	L
O -1	-8.055896	-0.009292	-2.773200	L
H -1	-8.739741	0.623700	-2.992079	L
H -1	-7.932513	0.087606	-1.828944	L
O -1	-1.447669	-4.028710	-0.670458	L
H -1	-1.018192	-4.872680	-0.530831	L
H -1	-0.734549	-3.430620	-0.894018	L
O -1	-8.972666	6.155608	3.894933	L
H -1	-8.219962	5.585680	3.737290	L
H -1	-9.208751	6.486568	3.028353	L
O -1	-6.837485	-0.642618	-5.435489	L
H -1	-6.893433	-0.647884	-4.479940	L
H -1	-6.663732	-1.554045	-5.670741	L
O -1	-3.327041	6.964484	3.823910	L
H -1	-3.423313	6.715297	2.904742	L
H -1	-2.895402	7.818417	3.797117	L
O -1	0.317635	-2.066230	-1.001831	L
H -1	1.270929	-2.068264	-1.088184	L
H -1	0.147863	-1.528312	-0.228492	L
O -1	-3.738760	-5.551006	-1.995051	L
H -1	-3.235442	-5.416952	-2.798127	L
H -1	-3.285363	-5.024210	-1.336910	L
O -1	-8.104014	-3.132568	0.315510	L
H -1	-8.206142	-2.697946	-0.531192	L
H -1	-8.591403	-3.951434	0.225272	L
O -1	-1.968881	0.853791	-5.989144	L
H -1	-1.049482	0.945796	-6.239089	L
H -1	-2.451677	0.952134	-6.809795	L
O -1	-5.916091	8.266763	2.717082	L
H -1	-5.555069	7.549645	3.238270	L
H -1	-6.258382	8.884418	3.363279	L
O -1	-5.534505	6.150675	5.914353	L
H -1	-5.758186	5.356786	6.400089	L
H -1	-4.912899	5.857303	5.248191	L
O -1	0.122445	3.100332	-3.776031	L
H -1	-0.279009	3.418484	-2.967424	L
H -1	-0.618049	2.887367	-4.343964	L
O -1	-3.918904	-1.918653	-6.776871	L
H -1	-4.552208	-1.975727	-6.061399	L
H -1	-3.149592	-1.506217	-6.384077	L
O -1	-3.362641	-2.303536	6.736642	L
H -1	-3.572252	-2.931952	6.045709	L
H -1	-4.063567	-1.653310	6.690364	L
O -1	-7.954826	0.147587	7.304074	L
H -1	-7.036264	-0.079441	7.159382	L
H -1	-8.428867	-0.673108	7.169998	L
O -1	-7.346237	-4.548746	5.102293	L
H -1	-7.377205	-4.142314	4.236217	L
H -1	-7.859124	-5.351553	5.009128	L
O -1	-5.957361	8.125005	-0.086106	L
H -1	-6.000675	8.011930	0.863404	L
H -1	-6.495277	8.897292	-0.260605	L
O -1	-10.386962	0.222064	1.088626	L
H -1	-10.494214	-0.487988	1.721520	L
H -1	-9.539125	0.051749	0.678263	L
O -1	-3.230219	8.222615	-0.702757	L
H -1	-3.001120	7.610281	-1.401894	L
H -1	-3.986091	7.823297	-0.272137	L
O -1	-8.862668	6.844899	-1.149467	L
H -1	-8.337737	6.172569	-1.583808	L
H -1	-9.375205	7.241492	-1.853919	L
O -1	-10.337684	-1.191895	5.016613	L
H -1	-9.383146	-1.223650	4.952729	L
H -1	-10.635665	-1.971212	4.547462	L
O -1	-2.429477	4.922967	5.229390	L
H -1	-1.513370	4.740396	5.438309	L
H -1	-2.426450	5.822753	4.902879	L
O -1	-6.206245	-4.996752	-3.059023	L
H -1	-5.510215	-5.266240	-2.459731	L
H -1	-6.968934	-5.506525	-2.785766	L
O -1	-10.777814	4.006355	-0.841995	L
H -1	-10.337802	3.455511	-0.194545	L
H -1	-10.065722	4.445111	-1.307451	L
O -1	-10.192779	-2.478376	2.363255	L
H -1	-11.098862	-2.422656	2.059709	L
H -1	-9.678657	-2.593963	1.564162	L
O -1	-6.589247	5.873098	-5.250986	L

O -1	-6.589247	5.873098	-5.250986	L
H -1	-6.957659	6.062248	-4.388010	L
H -1	-5.668797	5.673309	-5.080437	L
O -1	-9.711129	6.953470	1.511043	L
H -1	-9.867274	7.890808	1.626138	L
H -1	-9.277640	6.885062	0.660373	L
O -1	0.020461	-1.721032	3.655329	L
H -1	0.818937	-2.238288	3.760750	L
H -1	0.115946	-1.002188	4.280132	L
O -1	-11.652249	2.388517	3.535372	L
H -1	-11.449903	2.653068	4.432758	L
H -1	-10.798357	2.223816	3.135405	L
O -1	-6.265053	-3.415874	-5.062396	L
H -1	-6.792935	-3.859352	-5.726398	L
H -1	-6.340178	-3.970794	-4.286090	L
O -1	-5.714688	-6.907687	3.690358	L
H -1	-5.437191	-6.044104	3.384659	L
H -1	-5.049674	-7.160728	4.330637	L
O -1	-4.183697	0.387069	8.103622	L
H -1	-3.457715	0.983199	8.287500	L
H -1	-4.501501	0.122478	8.966886	L
O -1	0.815273	0.315123	1.951044	L
H -1	1.575464	0.765043	2.319709	L
H -1	0.607359	-0.367191	2.589362	L
O -1	-2.346493	-5.743993	5.398057	L
H -1	-2.929591	-4.999416	5.250292	L
H -1	-2.443547	-5.943738	6.329140	L
O -1	-9.764009	-2.192294	-2.270129	L
H -1	-8.876899	-2.074915	-2.609968	L
H -1	-10.178127	-1.336273	-2.379515	L
O -1	-10.243138	2.147349	6.247483	L
H -1	-9.992432	1.333068	6.683746	L
H -1	-9.852958	2.837176	6.784259	L
O -1	1.796957	3.362713	0.513430	L
H -1	0.978186	2.866959	0.522029	L
H -1	2.067546	3.363257	-0.404728	L
O -1	-0.215945	-4.739761	2.340328	L
H -1	-0.795324	-5.432745	2.023586	L
H -1	-0.116616	-4.918708	3.275392	L
O -1	-1.174233	8.288731	1.179929	L
H -1	-0.629109	9.041193	0.949989	L
H -1	-1.756737	8.175910	0.428801	L
O -1	0.364085	-4.940438	-4.095435	L
H -1	-0.559260	-4.689258	-4.071492	L
H -1	0.603866	-5.061127	-3.176647	L
O -1	-1.119546	8.407782	-2.948808	L
H -1	-1.858729	7.818003	-3.097136	L
H -1	-0.711135	8.501839	-3.809381	L
O -1	-7.828881	7.820180	5.794403	L
H -1	-7.023217	7.365376	6.039942	L
H -1	-8.266903	7.222309	5.188666	L
O -1	-6.289657	0.421757	-8.117578	L
H -1	-5.709160	-0.277449	-8.418187	L
H -1	-6.493537	0.191337	-7.211172	L
O -1	2.671606	-1.957102	-3.417232	L
H -1	3.097602	-2.456485	-4.113922	L
H -1	1.736105	-2.110709	-3.549421	L
O -1	-8.633155	-2.754016	7.043327	L
H -1	-8.267065	-3.470787	6.525205	L
H -1	-9.462425	-3.097462	7.375875	L
O -1	-9.556803	-0.433584	-5.244259	L
H -1	-8.641738	-0.383273	-5.520590	L
H -1	-9.890580	-1.227125	-5.662728	L
O -1	-7.098293	2.241086	9.487872	L
H -1	-7.445029	1.535595	8.941715	L
H -1	-7.874943	2.692483	9.818458	L
O -1	-9.963578	2.296157	-3.638776	L
H -1	-10.823318	1.946817	-3.404166	L
H -1	-10.129575	3.211876	-3.862685	L
O -1	-4.031908	-7.303912	0.745338	L
H -1	-3.383341	-7.103435	1.420172	L
H -1	-4.303015	-8.202640	0.932480	L
O -1	-6.077085	-6.164847	7.031857	L
H -1	-6.003385	-5.393197	6.470292	L
H -1	-5.615652	-5.922366	7.834674	L
O -1	-0.607235	-3.132615	7.224482	L
H -1	-1.146959	-2.357399	7.069654	L
H -1	0.152056	-3.014286	6.653775	L
O -1	0.608723	0.782479	4.877242	L
H -1	1.165837	1.426013	4.439385	L
H -1	0.552732	1.089643	5.782088	L
O -1	-12.031698	5.414356	1.171970	L
H -1	-11.653621	4.877763	0.475295	L
H -1	-11.342837	6.041237	1.392703	L
O -1	0.588663	0.896071	-6.764060	L
H -1	1.124866	0.247962	-6.307251	L
H -1	1.219021	1.435755	-7.241157	L
O -1	0.963654	5.175543	2.658173	L
H -1	1.330975	5.742758	1.980254	L
H -1	0.988025	4.298211	2.276170	L
O -1	-2.056881	-6.786513	2.848706	L
H -1	-2.393901	-6.630962	3.731006	L
H -1	-1.596537	-7.623379	2.911729	L
H -1	-6.957659	6.062248	-4.388010	L
H -1	-5.668797	5.673309	-5.080437	L
O -1	-9.711129	6.953470	1.511043	L
H -1	-9.867274	7.890808	1.626138	L
H -1	-9.277640	6.885062	0.660373	L
O -1	0.020461	-1.721032	3.655329	L
H -1	0.818937	-2.238288	3.760750	L
H -1	0.115946	-1.002188	4.280132	L
O -1	-11.652249	2.388517	3.535372	L
H -1	-11.449903	2.653068	4.432758	L
H -1	-10.798357	2.223816	3.135405	L
O -1	-6.265053	-3.415874	-5.062396	L
H -1	-6.792935	-3.859352	-5.726398	L
H -1	-6.340178	-3.970794	-4.286090	L
O -1	-5.714688	-6.907687	3.690358	L
H -1	-5.437191	-6.044104	3.384659	L
H -1	-5.049674	-7.160728	4.330637	L
O -1	-4.183697	0.387069	8.103622	L
H -1	-3.457715	0.983199	8.287500	L
H -1	-4.501501	0.122478	8.966886	L
O -1	0.815273	0.315123	1.951044	L
H -1	1.575464	0.765043	2.319709	L
H -1	0.607359	-0.367191	2.589362	L
O -1	-2.346493	-5.743993	5.398057	L
H -1	-2.929591	-4.999416	5.250292	L
H -1	-2.443547	-5.943738	6.329140	L
O -1	-9.764009	-2.192294	-2.270129	L
H -1	-8.876899	-2.074915	-2.609968	L
H -1	-10.178127	-1.336273	-2.379515	L
O -1	-10.243138	2.147349	6.247483	L
H -1	-9.992432	1.333068	6.683746	L
H -1	-9.852958	2.837176	6.784259	L
O -1	1.796957	3.362713	0.513430	L
H -1	0.978186	2.866959	0.522029	L
H -1	2.067546	3.363257	-0.404728	L
O -1	-0.215945	-4.739761	2.340328	L
H -1	-0.795324	-5.432745	2.023586	L
H -1	-0.116616	-4.918708	3.275392	L
O -1	-1.174233	8.288731	1.179929	L
H -1	-0.629109	9.041193	0.949989	L
H -1	-1.756737	8.175910	0.428801	L
O -1	0.364085	-4.940438	-4.095435	L
H -1	-0.559260	-4.689258	-4.071492	L
H -1	0.603866	-5.061127	-3.176647	L
O -1	-1.119546	8.407782	-2.948808	L
H -1	-1.858729	7.818003	-3.097136	L
H -1	-0.711135	8.501839	-3.809381	L
O -1	-7.828881	7.820180	5.794403	L
H -1	-7.023217	7.365376	6.039942	L
H -1	-8.266903	7.222309	5.188666	L
O -1	-6.289657	0.421757	-8.117578	L
H -1	-5.709160	-0.277449	-8.418187	L
H -1	-6.493537	0.191337	-7.211172	L
O -1	2.671606	-1.957102	-3.417232	L
H -1	3.097602	-2.456485	-4.113922	L
H -1	1.736105	-2.110709	-3.549421	L
O -1	-8.633155	-2.754016	7.043327	L
H -1	-8.267065	-3.470787	6.525205	L
H -1	-9.462425	-3.097462	7.375875	L
O -1	-9.556803	-0.433584	-5.244259	L
H -1	-8.641738	-0.383273	-5.520590	L
H -1	-9.890580	-1.227125	-5.662728	L
O -1	-7.098293	2.241086	9.487872	L
H -1	-7.445029	1.535595	8.941715	L
H -1	-7.874943	2.692483	9.818458	L
O -1	-9.963578	2.296157	-3.638776	L
H -1	-10.823318	1.946817	-3.404166	L
H -1	-10.129575	3.211876	-3.862685	L
O -1	-4.031908	-7.303912	0.745338	L
H -1	-3.383341	-7.103435	1.420172	L
H -1	-4.303015	-8.202640	0.932480	L
O -1	-6.077085	-6.164847	7.031857	L
H -1	-6.003385	-5.393197	6.470292	L
H -1	-5.615652	-5.922366	7.834674	L
O -1	-0.607235	-3.132615	7.224482	L
H -1	-1.146959	-2.357399	7.069654	L
H -1	0.152056	-3.014286	6.653775	L
O -1	0.608723	0.782479	4.877242	L
H -1	1.165837	1.426013	4.439385	L
H -1	0.552732	1.089643	5.782088	L
O -1	-12.031698	5.414356	1.171970	L
H -1	-11.653621	4.877763	0.475295	L
H -1	-11.342837	6.041237	1.392703	L
O -1	0.588663	0.896071	-6.764060	L
H -1	1.124866	0.247962	-6.307251	L
H -1	1.219021	1.435755	-7.241157	L
O -1	0.963654	5.175543	2.658173	L
H -1	1.330975	5.742758	1.980254	L
H -1	0.988025	4.298211	2.276170	L
O -1	-2.056881	-6.786513	2.848706	L
H -1	-2.393901	-6.630962	3.731006	L
H -1	-1.596537	-7.623379	2.911729	L
O -1	-0.344076	3.797584	-6.859831	L

O -1	-0.344076	3.797584	-6.859831	L
H -1	-0.287360	2.842071	-6.856493	L
H -1	-0.989955	3.997291	-7.537466	L
O -1	-2.653108	5.034183	8.622791	L
H -1	-2.929654	4.944689	7.710791	L
H -1	-3.379220	4.671810	9.130411	L
O -1	-4.180838	10.305145	-2.173661	L
H -1	-3.680199	10.605502	-2.932198	L
H -1	-3.744127	9.497760	-1.902288	L
O -1	-0.108398	5.829096	-4.676979	L
H -1	-0.176908	5.073726	-5.260891	L
H -1	-0.787183	6.430902	-4.982449	L
O -1	-10.851336	-4.235845	4.598794	L
H -1	-10.559398	-3.755322	3.824131	L
H -1	-10.251220	-4.979003	4.660508	L
O -1	-2.949910	0.556439	-8.505940	L
H -1	-3.737759	1.089222	-8.613960	L
H -1	-2.987691	-0.076056	-9.223405	L
O -1	-0.116251	-3.037015	-6.709343	L
H -1	-0.571675	-3.683923	-7.248166	L
H -1	0.796588	-3.325037	-6.708767	L
O -1	0.743543	7.311900	-1.363656	L
H -1	1.209640	6.827489	-2.045074	L
H -1	0.015243	7.730666	-1.822401	L
O -1	2.685105	2.915614	-2.102137	L
H -1	2.788193	1.975992	-2.252856	L
H -1	2.282287	3.244782	-2.905640	L
O -1	-0.315613	7.560688	3.839421	L
H -1	-0.845810	7.850674	3.097107	L
H -1	0.126010	6.771708	3.525235	L
O -1	-4.314000	10.656275	0.647153	L
H -1	-4.274357	9.805658	0.209990	L
H -1	-3.523128	11.111622	0.358340	L
O -1	-10.636034	5.635006	6.425494	L
H -1	-11.351596	6.270756	6.430647	L
H -1	-10.056022	5.932187	5.724422	L
O -1	2.338666	-4.592130	1.236409	L
H -1	1.397063	-4.650401	1.398337	L
H -1	2.435298	-3.842101	0.649595	L
O -1	0.353049	-5.980910	-1.081007	L
H -1	0.503925	-6.897920	-0.851743	L
H -1	1.230104	-5.613556	-1.190815	L
O -1	-6.238560	-2.372525	9.244047	L
H -1	-6.268215	-1.592533	9.798092	L
H -1	-6.908507	-2.219246	8.577784	L
O -1	-10.886512	-0.764901	7.622530	L
H -1	-10.807216	-0.964507	6.689737	L
H -1	-11.810476	-0.915143	7.822401	L
O -1	-6.338687	4.573264	-7.837655	L
H -1	-6.048795	5.411750	-8.197010	L
H -1	-6.623320	4.779299	-6.947281	L
O -1	0.392633	-4.908791	5.218893	L
H -1	-0.548500	-5.042977	5.330665	L
H -1	0.784983	-5.297450	6.000710	L
O -1	-4.257569	-8.544314	-1.880711	L
H -1	-4.772480	-9.039905	-1.243931	L
H -1	-4.150422	-7.680488	-1.482522	L
O -1	-12.940641	-0.669594	3.823293	L
H -1	-13.337628	0.189728	3.681171	L
H -1	-12.127757	-0.483653	4.293270	L
O -1	-4.459414	-1.866593	-9.542175	L
H -1	-3.976236	-1.866910	-8.715876	L
H -1	-4.861006	-2.734385	-9.585683	L
O -1	-13.177071	1.075416	0.593419	L
H -1	-13.490328	1.942566	0.850623	L
H -1	-12.223718	1.137306	0.652753	L
O -1	-3.113643	-2.589331	9.670857	L
H -1	-3.156806	-2.626500	8.715354	L
H -1	-3.989995	-2.314160	9.940154	L
O -1	2.768509	3.610778	-4.927078	L
H -1	1.849176	3.767457	-4.711414	L
H -1	2.938412	4.177392	-5.679615	L
O -1	-9.832226	9.537952	2.328289	L
H -1	-10.547588	9.581048	2.962822	L
H -1	-9.040350	9.480302	2.862930	L
O -1	-7.679820	-8.234504	2.046317	L
H -1	-8.302787	-7.624252	1.651681	L
H -1	-7.094643	-7.679901	2.562278	L
O -1	-4.247170	9.918107	5.702259	L
H -1	-4.678685	10.580564	5.162650	L
H -1	-3.766855	9.375092	5.077229	L
O -1	-5.079028	2.521003	-9.136212	L
H -1	-5.583386	1.876838	-8.639316	L
H -1	-5.374524	3.367513	-8.801049	L
O -1	3.088885	-2.282830	-0.541190	L
H -1	3.598743	-1.600885	-0.103894	L
H -1	3.200115	-2.104074	-1.474949	L
O -1	-1.779725	7.457822	-5.965801	L
H -1	-1.401397	8.098923	-6.567540	L
H -1	-2.713887	7.665936	-5.949650	L
O -1	-2.538999	9.827990	3.385931	L
H -1	-2.287619	10.728880	3.589492	L
H -1	-2.052473	9.617321	2.588973	L
H -1	-0.287360	2.842071	-6.856493	L
H -1	-0.989955	3.997291	-7.537466	L
O -1	-2.653108	5.034183	8.622791	L
H -1	-2.929654	4.944689	7.710791	L
H -1	-3.379220	4.671810	9.130411	L
O -1	-4.180838	10.305145	-2.173661	L
H -1	-3.680199	10.605502	-2.932198	L
H -1	-3.744127	9.497760	-1.902288	L
O -1	-0.108398	5.829096	-4.676979	L
H -1	-0.176908	5.073726	-5.260891	L
H -1	-0.787183	6.430902	-4.982449	L
O -1	-10.851336	-4.235845	4.598794	L
H -1	-10.559398	-3.755322	3.824131	L
H -1	-10.251220	-4.979003	4.660508	L
O -1	-2.949910	0.556439	-8.505940	L
H -1	-3.737759	1.089222	-8.613960	L
H -1	-2.987691	-0.076056	-9.223405	L
O -1	-0.116251	-3.037015	-6.709343	L
H -1	-0.571675	-3.683923	-7.248166	L
H -1	0.796588	-3.325037	-6.708767	L
O -1	0.743543	7.311900	-1.363656	L
H -1	1.209640	6.827489	-2.045074	L
H -1	0.015243	7.730666	-1.822401	L
O -1	2.685105	2.915614	-2.102137	L
H -1	2.788193	1.975992	-2.252856	L
H -1	2.282287	3.244782	-2.905640	L
O -1	-0.315613	7.560688	3.839421	L
H -1	-0.845810	7.850674	3.097107	L
H -1	0.126010	6.771708	3.525235	L
O -1	-4.314000	10.656275	0.647153	L
H -1	-4.274357	9.805658	0.209990	L
H -1	-3.523128	11.111622	0.358340	L
O -1	-10.636034	5.635006	6.425494	L
H -1	-11.351596	6.270756	6.430647	L
H -1	-10.056022	5.932187	5.724422	L
O -1	2.338666	-4.592130	1.236409	L
H -1	1.397063	-4.650401	1.398337	L
H -1	2.435298	-3.842101	0.649595	L
O -1	0.353049	-5.980910	-1.081007	L
H -1	0.503925	-6.897920	-0.851743	L
H -1	1.230104	-5.613556	-1.190815	L
O -1	-6.238560	-2.372525	9.244047	L
H -1	-6.268215	-1.592533	9.798092	L
H -1	-6.908507	-2.219246	8.577784	L
O -1	-10.886512	-0.764901	7.622530	L
H -1	-10.807216	-0.964507	6.689737	L
H -1	-11.810476	-0.915143	7.822401	L
O -1	-6.338687	4.573264	-7.837655	L
H -1	-6.048795	5.411750	-8.197010	L
H -1	-6.623320	4.779299	-6.947281	L
O -1	0.392633	-4.908791	5.218893	L
H -1	-0.548500	-5.042977	5.330665	L
H -1	0.784983	-5.297450	6.000710	L
O -1	-4.257569	-8.544314	-1.880711	L
H -1	-4.772480	-9.039905	-1.243931	L
H -1	-4.150422	-7.680488	-1.482522	L
O -1	-12.940641	-0.669594	3.823293	L
H -1	-13.337628	0.189728	3.681171	L
H -1	-12.127757	-0.483653	4.293270	L
O -1	-4.459414	-1.866593	-9.542175	L
H -1	-3.976236	-1.866910	-8.715876	L
H -1	-4.861006	-2.734385	-9.585683	L
O -1	-13.177071	1.075416	0.593419	L
H -1	-13.490328	1.942566	0.850623	L
H -1	-12.223718	1.137306	0.652753	L
O -1	-3.113643	-2.589331	9.670857	L
H -1	-3.156806	-2.626500	8.715354	L
H -1	-3.989995	-2.314160	9.940154	L
O -1	2.768509	3.610778	-4.927078	L
H -1	1.849176	3.767457	-4.711414	L
H -1	2.938412	4.177392	-5.679615	L
O -1	-9.832226	9.537952	2.328289	L
H -1	-10.547588	9.581048	2.962822	L
H -1	-9.040350	9.480302	2.862930	L
O -1	-7.679820	-8.234504	2.046317	L
H -1	-8.302787	-7.624252	1.651681	L
H -1	-7.094643	-7.679901	2.562278	L
O -1	-4.247170	9.918107	5.702259	L
H -1	-4.678685	10.580564	5.162650	L
H -1	-3.766855	9.375092	5.077229	L
O -1	-5.079028	2.521003	-9.136212	L
H -1	-5.583386	1.876838	-8.639316	L
H -1	-5.374524	3.367513	-8.801049	L
O -1	3.088885	-2.282830	-0.541190	L
H -1	3.598743	-1.600885	-0.103894	L
H -1	3.200115	-2.104074	-1.474949	L
O -1	-1.779725	7.457822	-5.965801	L
H -1	-1.401397	8.098923	-6.567540	L
H -1	-2.713887	7.665936	-5.949650	L
O -1	-2.538999	9.827990	3.385931	L
H -1	-2.287619	10.728880	3.589492	L
H -1	-2.052473	9.617321	2.588973	L
O -1	-4.961533	3.815108	10.205894	L

O	-1	-4.961533	3.815108	10.205894	L
H	-1	-5.597737	4.517542	10.071499	L
H	-1	-5.451749	3.011167	10.033850	L
O	-1	-7.023004	10.899405	0.814076	L
H	-1	-6.097459	10.723054	0.645258	L
H	-1	-7.215918	10.418876	1.619127	L
O	-1	-9.342576	-6.334641	0.485051	L
H	-1	-8.889847	-6.436143	-0.352186	L
H	-1	-10.191368	-5.956884	0.254651	L
O	-1	2.325621	6.842778	0.826542	L
H	-1	2.756076	7.656421	0.564006	L
H	-1	1.639563	6.712185	0.171941	L
O	-1	-2.907471	-5.927252	-6.243585	L
H	-1	-3.216126	-6.644946	-5.690521	L
H	-1	-3.637637	-5.750188	-6.836659	L
O	-1	0.459999	4.006335	6.430158	L
H	-1	0.167757	4.455747	7.223162	L
H	-1	0.439560	3.077237	6.659486	L
O	-1	-11.525476	-0.019671	-3.519743	L
H	-1	-12.204043	-0.580211	-3.896005	L
H	-1	-10.723737	-0.273548	-3.976901	L
O	-1	-0.744515	-0.500176	8.664038	L
H	-1	-1.333714	0.219297	8.890831	L
H	-1	-1.045246	-1.234486	9.199370	L
O	-1	-10.526407	4.661094	-4.848722	L
H	-1	-10.751534	4.606053	-5.777442	L
H	-1	-10.664444	5.580859	-4.622425	L
O	-1	-1.697199	-4.392313	-8.596422	L
H	-1	-2.326999	-4.443994	-7.877456	L
H	-1	-1.803657	-5.218143	-9.068548	L
O	-1	-12.926994	5.452995	-1.843869	L
H	-1	-12.091764	5.081502	-1.559939	L
H	-1	-13.580666	4.807885	-1.574095	L
O	-1	-13.076961	1.205136	6.950433	L
H	-1	-13.266477	0.389667	7.414465	L
H	-1	-12.133098	1.178179	6.793501	L
O	-1	2.219930	6.301880	-3.381010	L
H	-1	1.438171	6.159970	-3.914814	L
H	-1	2.913720	6.483413	-4.014992	L
O	-1	-9.435367	-0.263412	9.799546	L
H	-1	-9.891926	-0.391187	8.968006	L
H	-1	-9.878536	0.482103	10.204566	L
O	-1	-2.362918	3.886460	-8.570159	L
H	-1	-3.106205	3.339223	-8.823711	L
H	-1	-2.130438	4.360422	-9.368623	L
O	-1	-6.255968	6.067182	9.690212	L
H	-1	-6.607037	6.876304	10.062104	L
H	-1	-6.663596	6.003392	8.826499	L
O	-1	-0.567017	7.014442	6.471767	L
H	-1	-0.655002	7.421628	5.609972	L
H	-1	0.307819	7.265922	6.767826	L
O	-1	-10.106613	7.463817	-3.470223	L
H	-1	-10.609529	8.214719	-3.154858	L
H	-1	-9.963569	7.643929	-4.399378	L
O	-1	-8.108298	-6.884930	-2.492666	L
H	-1	-7.859193	-7.803090	-2.387025	L
H	-1	-9.006634	-6.840780	-2.165135	L
O	-1	-7.636387	10.411442	3.531235	L
H	-1	-6.766104	10.807130	3.483582	L
H	-1	-8.191792	11.097133	3.902160	L
O	-1	2.453980	-1.157707	-6.394144	L
H	-1	2.582401	-2.087938	-6.208642	L
H	-1	3.130461	-0.715153	-5.881550	L
O	-1	-13.856524	4.431179	3.116970	L
H	-1	-12.971965	4.142516	2.892338	L
H	-1	-14.381792	3.631172	3.099239	L
O	-1	-2.382480	1.743195	9.864051	L
H	-1	-2.229937	2.684865	9.942918	L
H	-1	-2.078024	1.380268	10.695809	L
O	-1	-4.276943	8.824453	-5.965325	L
H	-1	-3.746840	9.360785	-5.375775	L
H	-1	-5.169019	8.907464	-5.628365	L
O	-1	-11.243457	-4.406710	-1.372931	L
H	-1	-11.031448	-3.526372	-1.683237	L
H	-1	-11.816132	-4.264096	-0.619315	L
O	-1	0.673114	1.644394	7.572336	L
H	-1	0.346276	0.750971	7.678184	L
H	-1	0.946061	1.905849	8.451753	L
O	-1	-12.914062	-1.785888	1.279232	L
H	-1	-13.631203	-1.388705	0.785078	L
H	-1	-12.806746	-1.219641	2.043483	L
O	-1	-5.126795	0.220533	10.611160	L
H	-1	-4.529097	0.897477	10.928550	L
H	-1	-5.948391	0.390581	11.071915	L
O	-1	2.751618	-4.019691	-5.469411	L
H	-1	2.036869	-4.368910	-4.937047	L
H	-1	2.940459	-4.712778	-6.102026	L
O	-1	-7.217639	8.853751	-5.427317	L
H	-1	-7.557075	9.122551	-4.573642	L
H	-1	-7.961878	8.436133	-5.860833	L
O	-1	2.503432	2.750079	3.730138	L
H	-1	2.152572	3.630111	3.593489	L
H	-1	3.427120	2.819866	3.488971	L
H	-1	-5.597737	4.517542	10.071499	L
H	-1	-5.451749	3.011167	10.033850	L
O	-1	-7.023004	10.899405	0.814076	L
H	-1	-6.097459	10.723054	0.645258	L
H	-1	-7.215918	10.418876	1.619127	L
O	-1	-9.342576	-6.334641	0.485051	L
H	-1	-8.889847	-6.436143	-0.352186	L
H	-1	-10.191368	-5.956884	0.254651	L
O	-1	2.325621	6.842778	0.826542	L
H	-1	2.756076	7.656421	0.564006	L
H	-1	1.639563	6.712185	0.171941	L
O	-1	-2.907471	-5.927252	-6.243585	L
H	-1	-3.216126	-6.644946	-5.690521	L
H	-1	-3.637637	-5.750188	-6.836659	L
O	-1	0.459999	4.006335	6.430158	L
H	-1	0.167757	4.455747	7.223162	L
H	-1	0.439560	3.077237	6.659486	L
O	-1	-11.525476	-0.019671	-3.519743	L
H	-1	-12.204043	-0.580211	-3.896005	L
H	-1	-10.723737	-0.273548	-3.976901	L
O	-1	-0.744515	-0.500176	8.664038	L
H	-1	-1.333714	0.219297	8.890831	L
H	-1	-1.045246	-1.234486	9.199370	L
O	-1	-10.526407	4.661094	-4.848722	L
H	-1	-10.751534	4.606053	-5.777442	L
H	-1	-10.664444	5.580859	-4.622425	L
O	-1	-1.697199	-4.392313	-8.596422	L
H	-1	-2.326999	-4.443994	-7.877456	L
H	-1	-1.803657	-5.218143	-9.068548	L
O	-1	-12.926994	5.452995	-1.843869	L
H	-1	-12.091764	5.081502	-1.559939	L
H	-1	-13.580666	4.807885	-1.574095	L
O	-1	-13.076961	1.205136	6.950433	L
H	-1	-13.266477	0.389667	7.414465	L
H	-1	-12.133098	1.178179	6.793501	L
O	-1	2.219930	6.301880	-3.381010	L
H	-1	1.438171	6.159970	-3.914814	L
H	-1	2.913720	6.483413	-4.014992	L
O	-1	-9.435367	-0.263412	9.799546	L
H	-1	-9.891926	-0.391187	8.968006	L
H	-1	-9.878536	0.482103	10.204566	L
O	-1	-2.362918	3.886460	-8.570159	L
H	-1	-3.106205	3.339223	-8.823711	L
H	-1	-2.130438	4.360422	-9.368623	L
O	-1	-6.255968	6.067182	9.690212	L
H	-1	-6.607037	6.876304	10.062104	L
H	-1	-6.663596	6.003392	8.826499	L
O	-1	-0.567017	7.014442	6.471767	L
H	-1	-0.655002	7.421628	5.609972	L
H	-1	0.307819	7.265922	6.767826	L
O	-1	-10.106613	7.463817	-3.470223	L
H	-1	-10.609529	8.214719	-3.154858	L
H	-1	-9.963569	7.643929	-4.399378	L
O	-1	-8.108298	-6.884930	-2.492666	L
H	-1	-7.859193	-7.803090	-2.387025	L
H	-1	-9.006634	-6.840780	-2.165135	L
O	-1	-7.636387	10.411442	3.531235	L
H	-1	-6.766104	10.807130	3.483582	L
H	-1	-8.191792	11.097133	3.902160	L
O	-1	2.453980	-1.157707	-6.394144	L
H	-1	2.582401	-2.087938	-6.208642	L
H	-1	3.130461	-0.715153	-5.881550	L
O	-1	-13.856524	4.431179	3.116970	L
H	-1	-12.971965	4.142516	2.892338	L
H	-1	-14.381792	3.631172	3.099239	L
O	-1	-2.382480	1.743195	9.864051	L
H	-1	-2.229937	2.684865	9.942918	L
H	-1	-2.078024	1.380268	10.695809	L
O	-1	-4.276943	8.824453	-5.965325	L
H	-1	-3.746840	9.360785	-5.375775	L
H	-1	-5.169019	8.907464	-5.628365	L
O	-1	-11.243457	-4.406710	-1.372931	L
H	-1	-11.031448	-3.526372	-1.683237	L
H	-1	-11.816132	-4.264096	-0.619315	L
O	-1	0.673114	1.644394	7.572336	L
H	-1	0.346276	0.750971	7.678184	L
H	-1	0.946061	1.905849	8.451753	L
O	-1	-12.914062	-1.785888	1.279232	L
H	-1	-13.631203	-1.388705	0.785078	L
H	-1	-12.806746	-1.219641	2.043483	L
O	-1	-5.126795	0.220533	10.611160	L
H	-1	-4.529097	0.897477	10.928550	L
H	-1	-5.948391	0.390581	11.071915	L
O	-1	2.751618	-4.019691	-5.469411	L
H	-1	2.036869	-4.368910	-4.937047	L
H	-1	2.940459	-4.712778	-6.102026	L
O	-1	-7.217639	8.853751	-5.427317	L
H	-1	-7.557075	9.122551	-4.573642	L
H	-1	-7.961878	8.436133	-5.860833	L
O	-1	2.503432	2.750079	3.730138	L
H	-1	2.152572	3.630111	3.593489	L
H	-1	3.427120	2.819866	3.488971	L
O	-1	3.755517	1.662284	0.995878	L

O	-1	3.755517	1.662284	0.995878	L
H	-1	4.090975	1.507782	1.878957	L
H	-1	3.264453	2.480964	1.065524	L
O	-1	-9.518210	-6.129609	5.674003	L
H	-1	-10.090781	-5.786671	6.360143	L
H	-1	-9.150583	-6.931040	6.046547	L
O	-1	-5.156331	-5.911529	-7.630332	L
H	-1	-5.402926	-6.798908	-7.891061	L
H	-1	-5.893712	-5.602363	-7.104101	L
O	-1	-5.113356	8.785162	7.981834	L
H	-1	-4.952246	9.134998	7.105541	L
H	-1	-4.755658	7.897701	7.955449	L
O	-1	-7.734990	-4.892982	-6.732738	L
H	-1	-8.347211	-5.626801	-6.678637	L
H	-1	-8.079425	-4.344545	-7.437588	L
O	-1	-4.039032	-8.218723	5.341147	L
H	-1	-4.613890	-7.820273	5.994606	L
H	-1	-4.303777	-9.138276	5.317411	L
O	-1	-5.286420	11.717895	3.288729	L
H	-1	-5.440248	12.525229	2.798036	L
H	-1	-4.711348	11.200359	2.725097	L
O	-1	-5.529743	-5.060428	9.599726	L
H	-1	-5.348301	-4.143644	9.392797	L
H	-1	-5.167702	-5.184620	10.477071	L
O	-1	-2.569909	10.377539	-4.289101	L
H	-1	-1.969053	10.050060	-3.619802	L
H	-1	-2.009513	10.851652	-4.903433	L
O	-1	1.445763	-0.860430	-9.026871	L
H	-1	1.746752	0.031669	-9.199488	L
H	-1	1.519829	-0.959255	-8.077672	L
O	-1	2.426821	-2.471336	5.043767	L
H	-1	2.890956	-2.182549	5.829523	L
H	-1	2.820188	-3.317386	4.830007	L
O	-1	-9.819068	5.463696	9.343136	L
H	-1	-9.685166	5.709762	8.427847	L
H	-1	-10.193763	6.245222	9.749402	L
O	-1	-13.367207	4.344828	5.837475	L
H	-1	-13.562658	4.367048	4.900706	L
H	-1	-12.444843	4.593896	5.896158	L
O	-1	-1.447724	-5.320951	8.639396	L
H	-1	-1.815282	-4.909603	9.421653	L
H	-1	-0.987163	-4.612552	8.189633	L

H	-1	4.090975	1.507782	1.878957	L
H	-1	3.264453	2.480964	1.065524	L
O	-1	-9.518210	-6.129609	5.674003	L
H	-1	-10.090781	-5.786671	6.360143	L
H	-1	-9.150583	-6.931040	6.046547	L
O	-1	-5.156331	-5.911529	-7.630332	L
H	-1	-5.402926	-6.798908	-7.891061	L
H	-1	-5.893712	-5.602363	-7.104101	L
O	-1	-5.113356	8.785162	7.981834	L
H	-1	-4.952246	9.134998	7.105541	L
H	-1	-4.755658	7.897701	7.955449	L
O	-1	-7.734990	-4.892982	-6.732738	L
H	-1	-8.347211	-5.626801	-6.678637	L
H	-1	-8.079425	-4.344545	-7.437588	L
O	-1	-4.039032	-8.218723	5.341147	L
H	-1	-4.613890	-7.820273	5.994606	L
H	-1	-4.303777	-9.138276	5.317411	L
O	-1	-5.286420	11.717895	3.288729	L
H	-1	-5.440248	12.525229	2.798036	L
H	-1	-4.711348	11.200359	2.725097	L
O	-1	-5.529743	-5.060428	9.599726	L
H	-1	-5.348301	-4.143644	9.392797	L
H	-1	-5.167702	-5.184620	10.477071	L
O	-1	-2.569909	10.377539	-4.289101	L
H	-1	-1.969053	10.050060	-3.619802	L
H	-1	-2.009513	10.851652	-4.903433	L
O	-1	1.445763	-0.860430	-9.026871	L
H	-1	1.746752	0.031669	-9.199488	L
H	-1	1.519829	-0.959255	-8.077672	L
O	-1	2.426821	-2.471336	5.043767	L
H	-1	2.890956	-2.182549	5.829523	L
H	-1	2.820188	-3.317386	4.830007	L
O	-1	-9.819068	5.463696	9.343136	L
H	-1	-9.685166	5.709762	8.427847	L
H	-1	-10.193763	6.245222	9.749402	L
O	-1	-13.367207	4.344828	5.837475	L
H	-1	-13.562658	4.367048	4.900706	L
H	-1	-12.444843	4.593896	5.896158	L
O	-1	-1.447724	-5.320951	8.639396	L
H	-1	-1.815282	-4.909603	9.421653	L
H	-1	-0.987163	-4.612552	8.189633	L
